# Bioactive compound combinations from *Rhodiola tangutica* alleviate pulmonary vascular remodeling in high-altitude pulmonary hypertension rats through the PI3K–AKT pathway

**DOI:** 10.3389/fphar.2025.1582677

**Published:** 2025-05-02

**Authors:** Na Yang, Meiduo Huayu, Shanshan Su, Bin Hou, Zhanting Yang, Xingmei Nan, Zhanqiang Li

**Affiliations:** ^1^ Research Center for High Altitude Medicine, Qinghai University, Xining, China; ^2^ Xining Customs Technical Center, Key Laboratory of Food Safety Research in Qinghai Province, Xining, China; ^3^ Key Laboratory for High Altitude Medicine (Ministry of Education), Laboratory for High Altitude Medicine of Qinghai Province, Key Laboratory of Application and Foundation for High Altitude Medicine Research Qinghai Province (Qinghai-Utah Joint Research Key Lab for High Altitude Medicine), Qinghai University, Xining, China

**Keywords:** *Rhodiola tangutica*, bioactive equivalent combinatorial components, high-altitude pulmonary hypertension, pulmonary artery vascular remodeling, antioxidant, antiproliferation

## Abstract

**Background:**

Hypoxia-induced pulmonary vascular remodeling is central to the development of high-altitude pulmonary hypertension (HAPH). *Rhodiola tangutica* has traditionally been used to prevent chronic mountain sickness. Although its active fraction (ACRT) shows therapeutic potential for HAPH, the main pharmacodynamic substances remain unclear due to its complex composition.

**Aims:**

This study aimed to identify bioactive equivalent combinatorial components (BECCs) of ACRT that alleviate pulmonary vascular remodeling in HAPH rats and explore the underlying pharmacological mechanisms.

**Methods:**

Seventy adult Sprague–Dawley rats were divided into control, hypoxia, hypoxia + ACRT (150 mg/kg), hypoxia + BECCs (25, 50, and 100 mg/kg), and hypoxia + sildenafil (30 mg/kg) groups. An HAPH rat model was induced using a hypobaric hypoxia chamber simulating an altitude of 5,000 m. The effects of BECCs on pulmonary vascular remodeling in HAPH rats were evaluated based on hemodynamic indexes and histopathological changes, alongside antioxidant properties. Phosphoproteomics and Western blotting were performed to analyze AKT1-related protein expression in lung tissues. *In vitro*, 3% O_2_-induced pulmonary artery smooth muscle cell (PASMC) models were used to evaluate the anti-proliferative effects of BECCs and identify the dominant components. The underlying mechanisms were explored using Western blotting and a drug affinity responsive target stability (DARTS) assay to assess binding affinity.

**Results:**

HAPH rat models were successfully established, as evidenced by changes in physiological parameters. BECCs showed comparable efficacy to ACRT in restoring hemodynamic indexes and histopathological changes. Mechanistically, BECCs modulated AKT phosphorylation and related protein expression. *In vitro*, BECCs inhibited hypoxia-induced PASMC proliferation. Particularly, flavonoids (FLAs) within BECCs exhibited stronger anti-proliferative activity than other components, acting as the dominant contributors by regulating phosphatidylinositol-3 kinase (PI3K) rather than phosphoinositide-dependent protein kinase (PDPK) or mammalian target of rapamycin (mTOR) pathways to inhibit AKT phosphorylation. Among FLAs, eriodictyol and quercetin were found to inhibit PASMC proliferation by targeting PI3K.

**Conclusion:**

BECCs demonstrated comparable efficacy to ACRT in alleviating HAPH progression, reversing hypoxia-induced vascular remodeling, and inhibiting oxidative stress and PASMC proliferation by targeting the AKT protein. Flavonoids were identified as the key bioactive components contributing to the holistic effects of BECCs by regulating phosphatidylinositol-3 kinase/protein kinase B (PI3K/AKT) pathways. These findings could be extended to improve quality control and clarify the bioactive components of *R. tangutica* while inspiring development of combinatorial therapies for HAPH treatment.

## Graphical Abstract

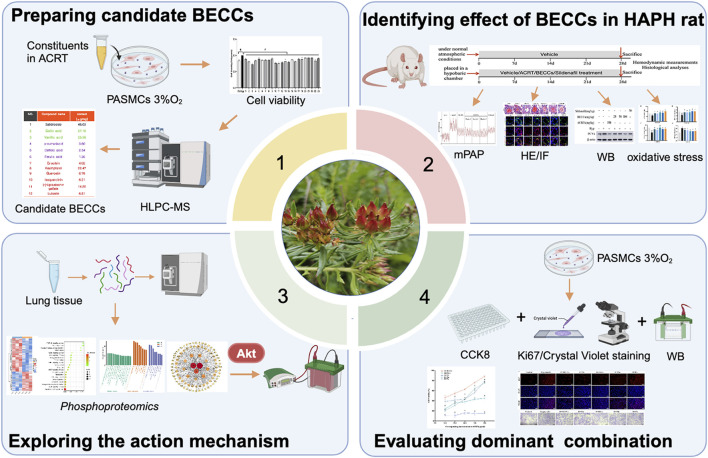

## Introduction

Pulmonary hypertension (PH) as a complex and fatal disorder leads to increased pulmonary vascular resistance, progressive pulmonary vascular remodeling, heart failure, and ultimately death (Boezio et al., 2017; Chen et al., 2019). Sustained exposure to chronic hypoxia above 2,500 m could induce high-altitude pulmonary hypertension (HAPH) in permanent residents (León-Velarde et al., 2005). The contraction and remodeling of pulmonary arteries are basic pathological features of HAPH, involving a range of cells, such as pulmonary artery smooth muscle cells (PASMCs), endothelial cells, and fibroblasts (Sydykov et al., 2021; Li et al., 2024). PASMCs are primary mediators of small pulmonary arterial remodeling, manifested as over-proliferation, migration, and resistance to apoptosis, which are key determinants in the progression of PH (Christou and Khalil, 2022). Furthermore, studies have shown that hypoxic exposure could induce oxidative stress, which was associated with vascular remodeling (Poyatos et al., 2023). As such, strategies aimed at improving vascular remodeling may offer therapeutic approaches for HAPH treatment.

*Rhodiola tangutica*, a widely recognized traditional Tibetan medicine with multiple components, is commonly used to prevent high-altitude sickness. Studies have shown many pharmacological activities of the compounds contained in *Rhodiola* on HAPH, such as the anti-proliferation of PASMCs (Zhang et al., 2024b), restoring endothelial cellular function (Ji et al., 2022), antioxidation (Polumackanycz et al., 2022), arresting cell cycle, and regulating autophagy and apoptosis (Liu et al., 2021; Tan et al., 2020). Nonetheless, these studies have typically focused on screening and identifying single active ingredients from *Rhodiola*, which could not fully represent the holistic therapeutic efficacy of the original medicine.

“Bioactive equivalent combinatorial components (BECCs)” refer to precisely defined combinations of constituents that collectively represent holistic pharmacological activity of the original herb, with clearly quantified proportions (Liu et al., 2014b). A previous study showed that a combination of 24 compounds retained the holistic properties of Qishen Yiqi (Zhang et al., 2018). Similarly, five compounds from Citrus reticulata “Chachi” peels were identified for antihyperlipidemic treatment (Xiao et al., 2022), and the BECCs of Angong Niuhuang pill were as effective as the original medicine in treating ischemic stroke (Zhang et al., 2025). Therefore, detecting BECCs of original medicines is crucial to uncover combinatorial therapeutic mechanisms and improve quality control of natural products.

Our previous study showed that the active fraction of *R. tangutica* (ACRT) alleviates HAPH and identified its main constituents (Zhang et al., 2022; Nan et al., 2018). **The** purpose of the present study was to explore whether a combination of 12 abundant bioactive compounds with anti-PASMC proliferation activity could exhibit comparable therapeutic efficacy to ACRT in HAPH rat models and qualify as bioactive equivalent combinatorial components. Accordingly, we prepared candidate BECCs, evaluated their bioactive equivalence *in vitro* and *in vivo*, investigated their anti-HAPH mechanism, identified key compounds, and clarified their pharmacological actions. This study provides evidence-based identification of the pharmacodynamic substances in *R. tangutica*. More importantly, it offers a widely applicable strategy for identifying combinatory compounds responsible for specific pharmacological activities in herbal medicines.

## Materials and methods

### Materials

Chemical compounds of BECCs: gallic acid (purity > 98%, LOT: J1120A) was obtained from Dalian Meilun Biotechnology Co., Ltd. Vanillic acid (BR, 98%, LOT:F081S206771), caffeic acid (AR, 98%, LOT: M28HS183194), p-coumaric acid (AR, 98%, LOT: L13O7Z22606), kaempferol (purity 95%, LOT: J09GS153993), isoquercitrin (purity 98%, LOT: X29O11Y128970), ferulic acid (purity > 98%, LOT: H27J7L16718), and luteolin (HPLC > 98%, LOT: C29N10Q104574) were purchased from Shanghai Yuanye Biotechnology Co., Ltd. Quercetin (purity 97%, LOT: C12819180), epicatechin (purity 95%, LOT: C15754541), eriodictyol (purity 98%, LOT: C16253744), and salidroside (purity 98%, LOT: C14565287) were acquired from Shanghai Macklin Biochemical Technology Co., Ltd. Their chemical structures are shown in Supplementary Figure S1. Sildenafil (LOT: X23A8Y42189), SC79 (AKT activator, LOT: F08J11F115126), 740Y-P [phosphatidylinositol-3 kinase (PI3K) activator, LOT: M121S214979], PS210 [phosphoinositide-dependent protein kinase 1 (PDPK1) activator, LOT: S15GS161210], MHY1485 [mammalian target of rapamycin (mTOR) activator, LOT: J29J8Y38900], and LY294002 (PI3K inhibitor, LOT: Z22M7H15080) were acquired from Yuanye Biotechnology, Shanghai, China. ACRT was obtained as previously reported (Nan et al., 2018). Candidate BECCs were prepared by mixing reference compounds in proportions according to their concentrations in ACRT.

Anti-AKT1 (BM4390), anti-PI3K (M01091-1), anti-PDPK1 (PB0777), and anti-mTORC2 (BM4182) were purchased from Wuhan Boster Biotechnology Co., Ltd. Anti-phosphoserine mTORC2 (#AF3308), anti-phosphoserine PDPK1 (#AF3018), and anti-phosphoserine PI3K (#AF3241) were acquired from Affinity Technology Co., Ltd. (Jiangsu, Zhejiang, China). Anti-GSK-3β (A22665), anti-phosphoserine GSK-3β (AP1341), anti-cyclin-dependent kinase-4 (CDK4, A11136), anti-cyclin D1 (A19038), anti-p27kip1 (A0290), anti-proliferating cell nuclear antigen (PCNA) (A12427), anti-cyclin A2 (A19036), anti-Ki67 (A23722), anti-α-smooth muscle actin (A17910), and ABflo^R^ secondary antibody were obtained from Wuhan ABclonal Technology Co., Ltd. Anti-phosphoserine AKT1 (Ser473) (CY6569) was sourced from Abways Technology Co., Ltd.(Shanghai, China). Anti-CDK2, (10122-1-AP), anti-β-actin, and secondary antibodies were acquired by Wuhan Proteintech Technology Co., Ltd.

### Candidate BECCs preparation

Based on our previous research (Nan et al., 2018), we conducted active ingredient screening on the main components of ACRT in 3% O_2_-induced PASMC models and found that almost all components contributed to the overall efficacy of ACRT by inhibiting the proliferation of PASMCs induced by hypoxia, but no single component could account for the whole therapeutic efficacy of ACRT (Supplementary Figure S1). Therefore, we considered that the concentration of each compound is a critical factor as it may be closely associated with the overall efficacy. We determined their concentration and selected active ingredients exceeding 0.1% (1/1,000) of the total as candidate components for BECC formulation. As a result, a combination of 12 bioactive compounds (listed in Supplementary Table S1) was designated as the candidate BECCs of ACRT, and their bioactivities were further validated *in vivo* and *in vitro*.

### Animals

The animal experimental protocol was approved by the Animal Ethics Committee of Qinghai University (Ethics number: 2023-004) and conducted in accordance with the guidelines of the National Academy of Sciences of the National Institutes of Health (NIH). Seventy Sprague –Dawley male rats (weighing 140 ± 10 g, 6–8 weeks old) supplied by the Beijing Vital River Laboratories [approval NO. SCXK (Jing) 2022–0063] were randomly assigned into seven groups (10 rats per group), namely, control, hypoxia (H), H + ACRT (150 mg/kg), H + BECCs (25, 50, and 100 mg/kg), and H + sildenafil (30 mg/kg). ACRT, BECCs, and sildenafil were suspended in 0.5% carboxymethylcellulose sodium (CMC-Na) and administered intragastrically for 28 days. The control and hypoxia groups were given an equal amount of CMC-Na. The dosage adopted was primarily based on our previous study (Nan et al., 2018). Meanwhile, rats in the hypoxia groups were housed in a hypobaric chamber (DYC-300, Guizhou Fenglei Oxygen Chamber Co., Ltd., Guizhou, China), which was adjusted to an altitude of 5,000 m, with an atmospheric pressure of 52.9 kPa and an oxygen partial pressure of 42 mmHg.

### Hemodynamic measurements

Following 28 days of exposure to hypoxia or normoxia, all rats were weighed and given an intraperitoneal injection of 20% urethane (1.0 g/kg) to induce anesthesia. Then, right cardiac catheterization was conducted to measure mean pulmonary artery pressure (mPAP). A heparin-coated polystyrene microcatheter was used to establish a connection between the pressure transducer (Model TSD104A; MP150 BIOPAC Systems, United States) and the biological signal acquisition system. Then, the right external jugular vein was exposed, and the catheter was inserted in the right internal jugular vein and carefully advanced into the right ventricle (RV). Under real-time guidance, it was then maneuvered through the right ventricular outflow tract (RVOT), traversed the pulmonary valve, and positioned within the main pulmonary artery (PA) to obtain direct mPAP measurements. The accurate position of the inserted catheter was determined by the waveform displayed on the biological signal sensing system (MP-150, BIOPAC Systems, United States). Then, the blood sample was obtained to perform a routine blood analysis. Subsequently, organs, including the heart, liver, lungs, kidney, and spleen, were collected, weighed, and used to calculate the organ index. The heart was dissected and separated into the septum (S), left ventricle (LV), and RV which were then weighed to assess indices of RV hypertrophy, including the ratio of RV weight to body weight (RV/BW) and the ratio of RV weight to the weight of LV and spleen RV/(LV + S).

### Histological morphological analysis

The left lung lobe tissues were fixed, dehydrated, embedded, sectioned, and finally stained using the hematoxylin and eosin (HE) method to evaluate pulmonary vascular remodeling (n = 3/group). Then, arteries with diameters less than 100 µm were gauged using Image-Pro Plus 6.0 version (Media Cybernetics, United States). The indicators were quantified at ×400 magnification, including the proportion of the wall area (WA%), the proportion of the pulmonary artery lumen area to the cross-sectional area (LA%), and the proportion of wall thickness (WT%) (Chen et al., 2022).

### Immunofluorescent staining

The lower left lung tissues (n = 3/group) were embedded with OTC, then sectioned into slices, and incubated with the antibodies of α-smooth muscle actin (α-SMA) (1:200) and PCNA (1:200) at 4°C. Subsequently, on the next day, the secondary fluorescent antibody was cultured for one more hour at 37°C under darkness. The nuclei were counterstained with DAPI. The section images were obtained using a co-focus laser scanning microscope (FV1000, Olympus, Japan).

### Measurement of oxidative stress

The parameters of oxidative stress in lung tissue and PASMCs were assessed according to the manufacturer’s instructions (Jiancheng Bioengineering Institute, Nanjing, China), including superoxide dismutase (SOD) and glutathione peroxidase (GSH-Px) activities, along with glutathione (GSH) and malondialdehyde (MDA) contents.

### Phosphoproteomics of lung tissue from HAPH rats

Mass spectrometry analysis was performed on the processed peptide samples to obtain mass-spectrometry data, which were subsequently transformed into protein identification and quantified. Then, differential proteins were screened, and bioinformatics analyses were performed. Gene Ontology (GO) analysis was carried out using eggNOG-mapper software (http://eggnog5.embl.de/#/app/home). KAAS software (http://www.genome.jp/kegg/kaas/) was used to conduct the Kyoto Encyclopedia of Genes and Genomes (KEGG) pathway analyses; both were visualized using an online platform (https://www.bioinformatics.com.cn). Protein–protein interaction (PPI) analysis was performed using the STRING database. Cytoscape 3.8.2 was used for visualization.

### Western blot

Total protein was extracted from cells or tissues (50 mg) using lysate. Following the measurement of the concentration, samples were isolated using SDS-PAGE and relocated to PVDF membranes, which were blocked and then incubated with the following primary antibodies: p-AKT1 (S473) (1:1,000), AKT1 (1:1,000), GSK3β (1:2,000), p-GSK3β (1:800), PCNA (1:2,000), P27 (1:1,000), cyclin D1 (1:1,500), cyclin A2 (1:2,000), CDK2 (1:2,000), CDK4 (1:2,000), PI3K (1:1,200), p-PI3K (1:1,200), PDK1 (1:800), p-PDK1 (1:800), mTOR (1:1,500), p-mTOR (1:1,500), and β-actin (1:10,000) overnight at 4°C. The secondary antibody (1:5,000) was then incubated for one more hour. Chemiluminescent reagents (Boster, China) and ImageJ software were used to display and quantify immunoblots.

### Primary PASMC isolation and culture

Male SD rats (100 ± 10 g) were euthanized by cervical dislocation. Pulmonary arteries were rapidly isolated from surrounding tissues on an ultraclean workbench, and the endothelium and epithelium were carefully removed from the vessels. Isolated pulmonary arterioles were cut into pieces before being digested with 0.3% collagenase II. Then, deposition after centrifugation was collected carefully. The primary PASMCs were treated in Dulbecco’s modified Eagle Medium (DMEM) F-12 containing 20% fetal bovine serum (FBS) and cultured in a 5% CO_2_ incubator. PASMCs were identified via immuno-histochemical staining with α-SMA. All experiments were conducted using cells at passages 2–6.

### Cell viability assay

The 12 compounds in BECCs were structurally classified into four chemical families, namely, phenolic acids (PAs, including gallic acid and vanillic acid), flavonoids (FLAs, including eriocitrin, kaempferol, quercetin, isoquercitrin, epicatechin, and luteolin), phenylethanoid glycosides (PeGs, including salidroside), and phenylpropanoids (PhEs, including p-coumaric acid, ferulic acid, and caffeic acid), to identify which group of constituents was responsible for the anti-proliferative activity of BECCs (Supplementary Table S1). PASMCs were cultivated at approximately 6 × 10^3^ cells per well in 96-well plates. After adherence, cells were serum-starved for 12 h. Then, cells were treated with varying concentrations of BECCs (0–250 μg/mL). Twelve compounds comprising BECCs (0–1,000 μM) and PAs, FLAs, PeGs, and PhEs (0–250 μg/mL) were used under normoxia or 3% O_2_ conditions for 24 h. Subsequently, the CCK-8 reagent (Elabscience Biotechnology Co., Ltd, Wuhan, China) was added and incubated. One hour later, absorbance was measured to determine the 50% inhibitory concentration (IC_50_).

### Measurement of cell proliferation

A density of 2 × 10^4^ PASMCs per well was planted into 12-well plates. After starvation, PASMCs were treated with BECCs (50 μg/mL) or compound combinations, including PeGs (14.3 μg/mL), PAs (15.5 μg/mL), PhEs (2.2 μg/mL), or FLAsa (18.0 μg/mL), and key bioactive compounds, including eriocitrin (ERI, 11 μM) and quercetin (QCT, 18 μM), under hypoxia conditions for 24 h. The concentrations of each group and compound were equivalent to their corresponding concentrations in 50 μg/mL of BECCs (Supplementary Table S1). After termination of drug intervention, crystal violet was used to stain PASMCs, which were then fixed with paraformaldehyde. Subsequently, cells were washed with flowing water and photographed under a light microscope. For immunofluorescent staining, PASMCs were similarly preserved with paraformaldehyde, then permeabilized, blocked, and incubated with the Ki67 antibody (1:200) at 4°C. On the following day, cells were incubated with the secondary fluorescent antibody (1:200) protected from light at room temperature. DAPI was used to stain the nuclei. The dosage selected was mainly based on the relevant content in BECCs and IC_50_ concentration. Images were acquired through laser scanning confocal microscopy (ZEISS Axio Vert A1, Germany).

### Drug affinity responsive target stability analysis

M-PER lysis solution (LOT: YH371252, Thermo Fisher Scientific, United States) was used to lyse PASMCs (7 × 10^7^) on ice. Then, the sample obtained following centrifugation was adjusted to the appropriate concentration using the 1 × TNC buffer. The mixture was divided into two groups and then treated with either BECCs (50 μg/mL) or 1% DMSO, respectively. Following 1 h of incubation, the two groups were further subdivided into different subgroups and then exposed to varying concentrations of pronase for 25 min under 37°C. Different concentrations of pronase were pre-prepared with the TNC buffer (Zhao et al., 2023). After the termination of proteolysis, a loading buffer was added. Then, the samples were boiled. The expressions of proteins were analyzed using the Western blotting test. For eriocitrin (ERI, 11 μM) and quercetin (QCT, 18 μM), the same method was used. The drug dosage was determined based on the IC_50_ concentration, the corresponding concentration of each compound, and previous research (Morales-Cano et al., 2014; He et al., 2015; Gao et al., 2024).

### Statistical analysis

We analyzed all data using GraphPad Prism 10.0 (GraphPad Software Inc., San Diego, United States). Data with normal distribution were shown as the mean ± standard deviation (SD). The two-group data were analyzed using an unpaired t-test. Multiple-group statistical significance was determined using one-way ANOVA. For all comparisons, statistical significance was set at *p* < 0.05.

## Results

### Bioactive compound combinations ameliorated HAPH and pulmonary vascular remodeling

**FIGURE 1:**
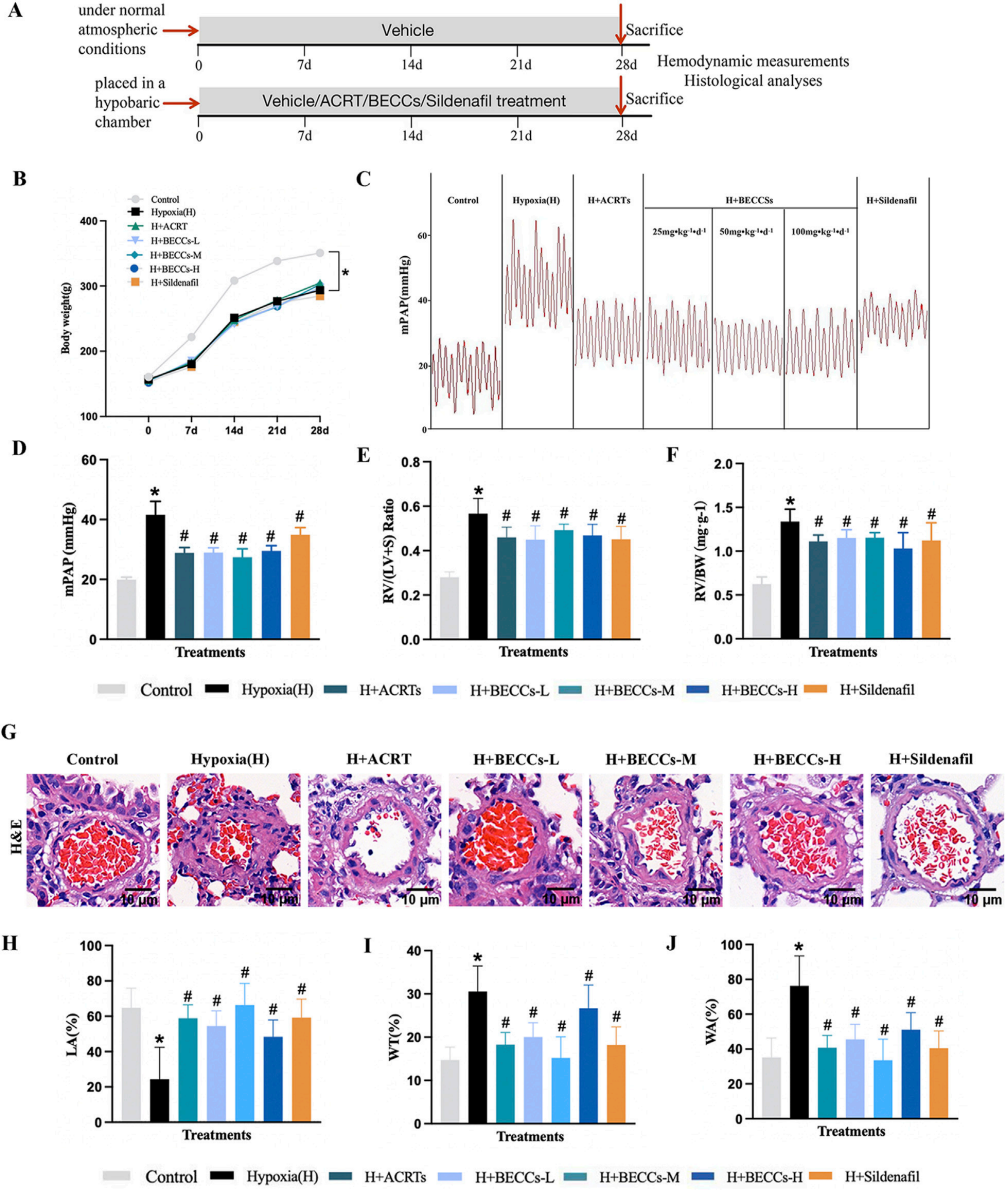
Candidate BECCs ameliorate HAPH and pulmonary vascular remodeling in rats. **(A)** Experimental strategy of modeling and treatments. **(B)** Changes in weight from seven groups (n = 10). **(C)** Representative mPAP image wave (n = 7). **(D)** Quantitative evaluation of mPAP (n = 7). **(E)** RV/(LV + S) ratio (n = 5–8). **(F)** RV/BW ratio (n = 5–8). **(G)** Representative images of the rat pulmonary artery by HE staining (n = 3, ×400 magnification, outer diameter 20–100 μm, scale bar = 10 μm). **(H)** LA% of pulmonary arteries. **(I)** WT% of pulmonary arteries. **(J)** WA% of pulmonary arteries. H Hypoxia. ACRT, bioactive fraction from Rhodiola—150 mg/kg; BECCs-L, candidate bioactive equivalent combinatorial components—25 mg/kg; BECCs-M, candidate bioactive equivalent combinatorial components—50 mg/kg; BECCs-H, candidate bioactive equivalent combinatorial components—100 mg/kg; Sildenafil: 30 mg/kg. All data are represented as the mean ± SD. *p < 0.05 vs. control group, ^#^ *p* < 0.05 vs. hypoxia group, and *p* < 0.05 vs. ACRT group.

An HAPH model was constructed using a hypobaric chamber (Figure 1A). First, candidate BECC-mediated changes in the body weight of experimental rats were assessed. The data showed no differences at baseline. After 28 days, rats under hypoxic conditions had noticeably lower weight than those in the control group (*p* < 0.05, Figure 1B), whereas no difference was observed between the hypoxic and treatment groups. Simultaneously, we assessed the visceral index, which revealed that the organ-to-body weight ratios, including the spleen, lungs, and heart, were elevated in the hypoxia group compared to those in the normoxia group, while liver or kidney indices were decreased. Pretreatment with ACRT or candidate BECCs effectively mitigated the liver and lung index alterations induced by hypoxia (*p* < 0.05, Supplementary Table S2). Notably, ACRT and candidate BECCs showed no statistical differences (p > 0.05), indicating equivalent efficacy.

We further tested the impact of candidate BECCs on mPAP and right heart hypertrophy. Results showed that mPAP, RV/(LV + S), and RV/BW in the hypoxia group were markedly elevated compared to those in the normoxia group. These changes were attenuated by ACRT, candidate BECCs, or sildenafil treatment (*p* < 0.05, Figures 1C–F). Subsequently, we identified whether candidate BECCs affected hemodynamics in HAPH rats. Our results indicated that red blood cell (RBC), hemoglobin (HGB), hematocrit (HCT), and white blood cell (WBC) levels were significantly increased under hypoxia, while the platelet (PLT) level was reduced compared to that in the normoxia group (*p* < 0.05, Supplementary Table S3). ACRT or candidate BECC mediation remarkably restored the hypoxia-induced changes. However, sildenafil intervention only affected the alterations in PLTs (*p* < 0.05, Supplementary Table S3). Interestingly, no differences were found in mPAP, RV/(LV + S), RV/BW, and hematological indicators between the ACRT and candidate BECC groups (*p* > 0.05), which suggested that the candidate BECCs could be a bioactive equivalent to the original ACRT.

The HE stains of the vascular diameters less than 100 μm revealed obvious hypoxia-induced vascular remodeling, manifesting as a noticeable elevation in the proportion of the wall area (WA%), along with the proportion of wall thickness (WT%), and a considerable decrease in the proportion of the pulmonary artery lumen area to the cross-sectional area (LA%). These changes were reversed in the presence of ACRT, candidate BECCs, or sildenafil (*p* < 0.05, Figures 1G–J). Interestingly, no difference was found in WA%, WT%, and LA% between the ACRT group and candidate BECC group (*p* > 0.05), which suggested that the candidate BECCs could be a bioactive equivalent to the original ACRT. Thus, the combination of 12 abundant compounds was considered the BECCs of ACRT against vascular remodeling in HAPH rats.

### BECCs attenuated the hypoxia-induced proliferation of PASMCs and oxidative stress in rats

**FIGURE 2:**
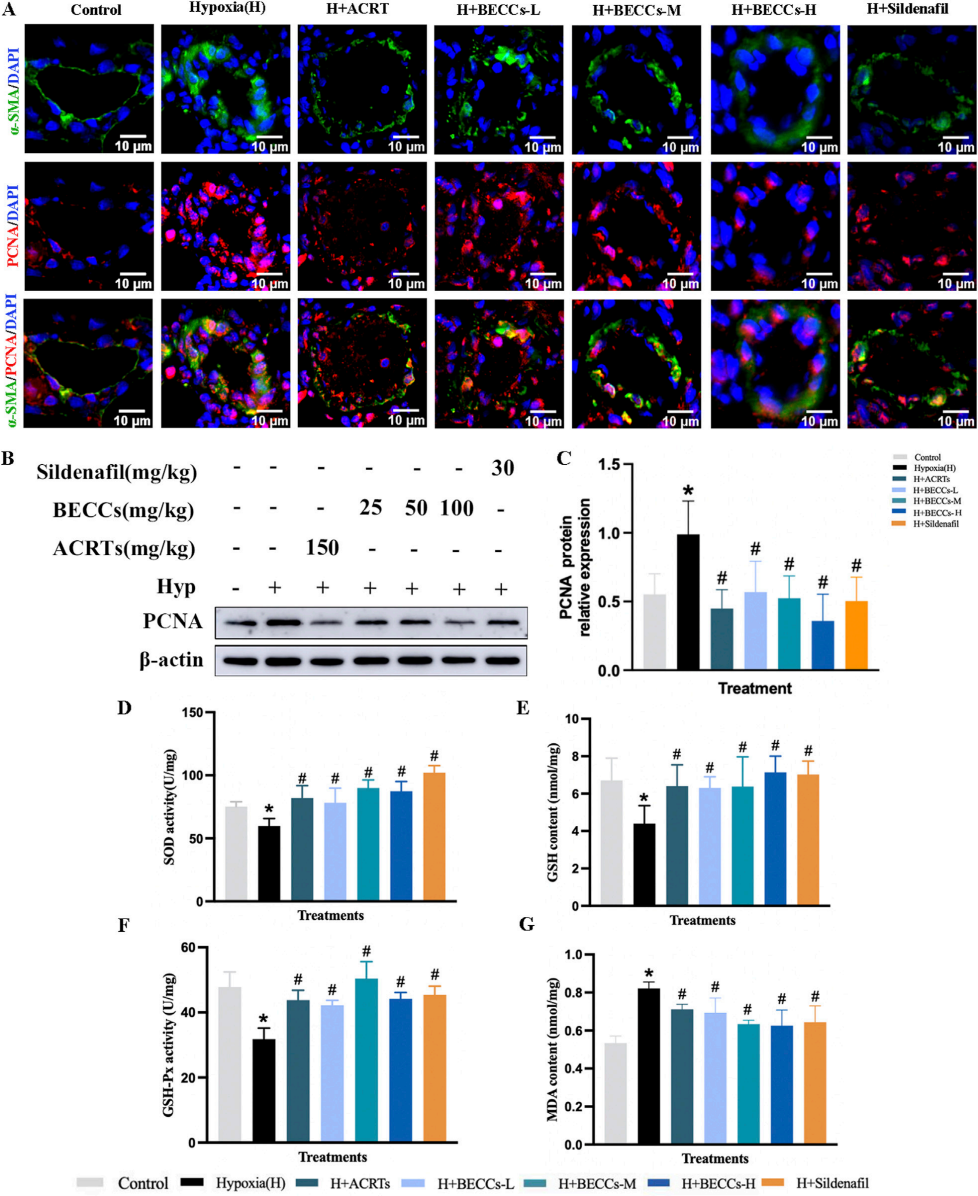
BECCs suppress hypoxia-induced PASMC proliferation and oxidative stress in rats. **(A)** Representative immunofluorescence staining images (n = 3, ×400 magnification, outer diameter 20–100 μm, scale bar = 10 μm). **(B)** Primary bands of PCNA by Western blots. **(C)** Quantitative data (n = 5). **(D–G)** Quantitative evaluation of SOD and GSH-Px activities and GSH and MDA contents in the lung tissue of HAPH rats. All data are represented as the mean ± SD. *p < 0.05 vs. control group, ^#^ *p* < 0.05 vs. hypoxia group, and *p* < 0.05 vs. ACRT group.

For further assessing the role of BECCs in pulmonary arterial remodeling, whether or not associated with the proliferation of PASMCs, α-SMA and proliferating cell nuclear antigen (PCNA) antibodies were stained via immunofluorescence. As shown in Figure 2A, α-SMA and PCNA expression levels around pulmonary vessels were significantly elevated under hypoxia compared to those in the normoxia group, which were evidently downregulated by ACRT, BECC, or sildenafil treatment. Consistently, the protein level of PCNA in the lung tissue was higher in the hypoxia group than that under normoxic conditions, which was decreased by ACRT, BECC, or sildenafil treatment (*p* < 0.05, Figures 2B, C). Importantly, no statistical differences were observed in PCNA expression and relevant histopathological indicators between ACRT and BECC treatments, indicating that BECCs could explain the antiproliferative efficacy of ACRT (*p* > 0.05).

Furthermore, compared to the normoxia group, hypoxia exposure significantly decreased the levels of SOD, GSH-Px, and GSH, accompanied by an increased content of MDA in the lung tissue of HAPH rats (*p* < 0.05, Figures 2D–G). ACRT, BECC, and sildenafil treatments reversed these changes. Additionally, compared with the ACRT group, BECCs demonstrated nearly identical antioxidant effects (*p* > 0.05, Figures 2D–G).

### Phosphoproteomic profiling in HAPH rat lung tissues

**FIGURE 3:**
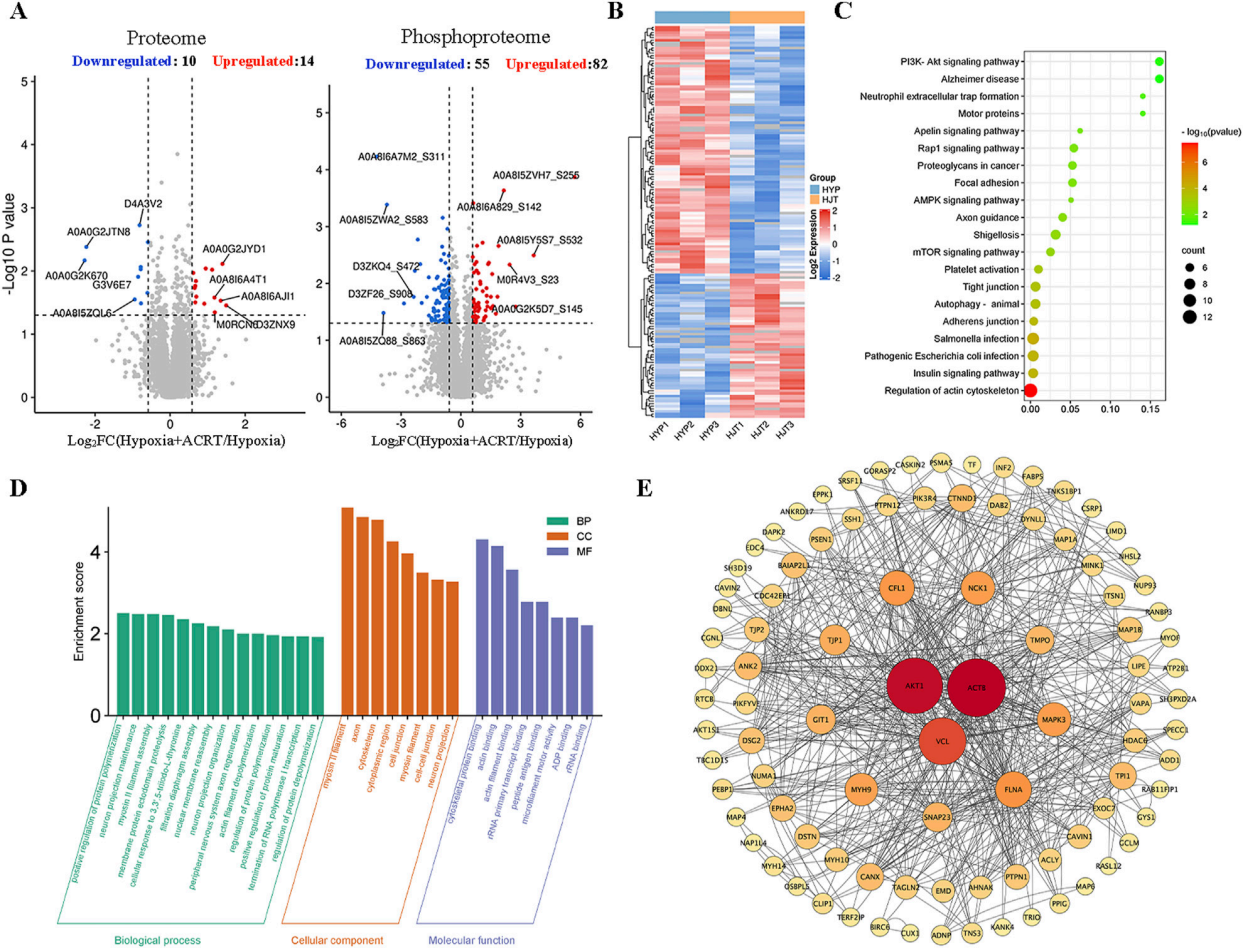
Quantified phosphopeptide analysis. **(A)** Distribution of quantified proteins in the volcano plot. **(B)** Visual heatmap of differential modification sites. **(C)** Pathway enrichment analysis. **(D)** GO enrichment analysis. **(E)** PPI network analysis of all significantly altered proteins in phosphopeptides. The nodes in the inner circle are the core targets in the network.

In order to elucidate the potential mechanism of BECCs in preventing HAPH, lung tissues of hypoxia and ACRT groups were subjected to proteomic and phosphoproteomic analyses. In the proteome research, a series of 5,397 proteins were determined, among which 5,395 proteins were quantified (Supplementary Table S4). Statistical analysis indicated that 24 proteins were remarkably altered (|log2 (fold change)|> log1.5), including 10 downregulated and 14 upregulated proteins (*p* < 0.05, Figure 3A). For the phosphoproteomic study, 10,575 proteins were discovered in all, wherein 2,056 proteins were quantified (Supplementary Table S5). Statistical analysis illustrated 82 downregulated and 55 upregulated phosphoproteins (Figure 3A). As the phosphorylation of multiple proteins affects the progression of PH, we performed a well-rounded signaling pathway and PPI analysis of differentially expressed phosphoproteins. To provide a general understanding of all these changes, the significantly altered protein abundances are shown in a heatmap (Figure 3B). We further conducted KEGG pathway enrichment analyses. The top 20 targets were enriched in signaling pathways such as the mTOR pathway, insulin pathway, AMPK pathway, and phosphatidylinositol-3 kinase/protein kinase B (PI3K/AKT) pathway (Figure 3C). The GO analysis showed that ACRT treatment in HAPH primarily participated in biological processes including protein polymerization, maturation, and depolymerization. These molecular targets were functionally associated with cytoskeletal protein binding, actin binding, and rRNA transcript binding, while being predominantly localized in cellular compartments such as the cytoplasmic region, cell junctions, cytoskeleton, and neuronal projections (Figure 3D). Thereafter, a PPI network was established to better elaborate the connections between these significantly altered proteins under hypoxia (Figure 3E). Notably, AKT1, ACTB, and VCL had a higher degree. Therefore, based on the aforementioned studies and the critical role of AKT phosphorylation in proliferation, we hypothesized that it might also be the primary target of ACRT and BECCs in HAPH.

### BECC treatment decreased phosphorylation levels of AKT1 and GSK3β in HAPH rats

**FIGURE 4:**
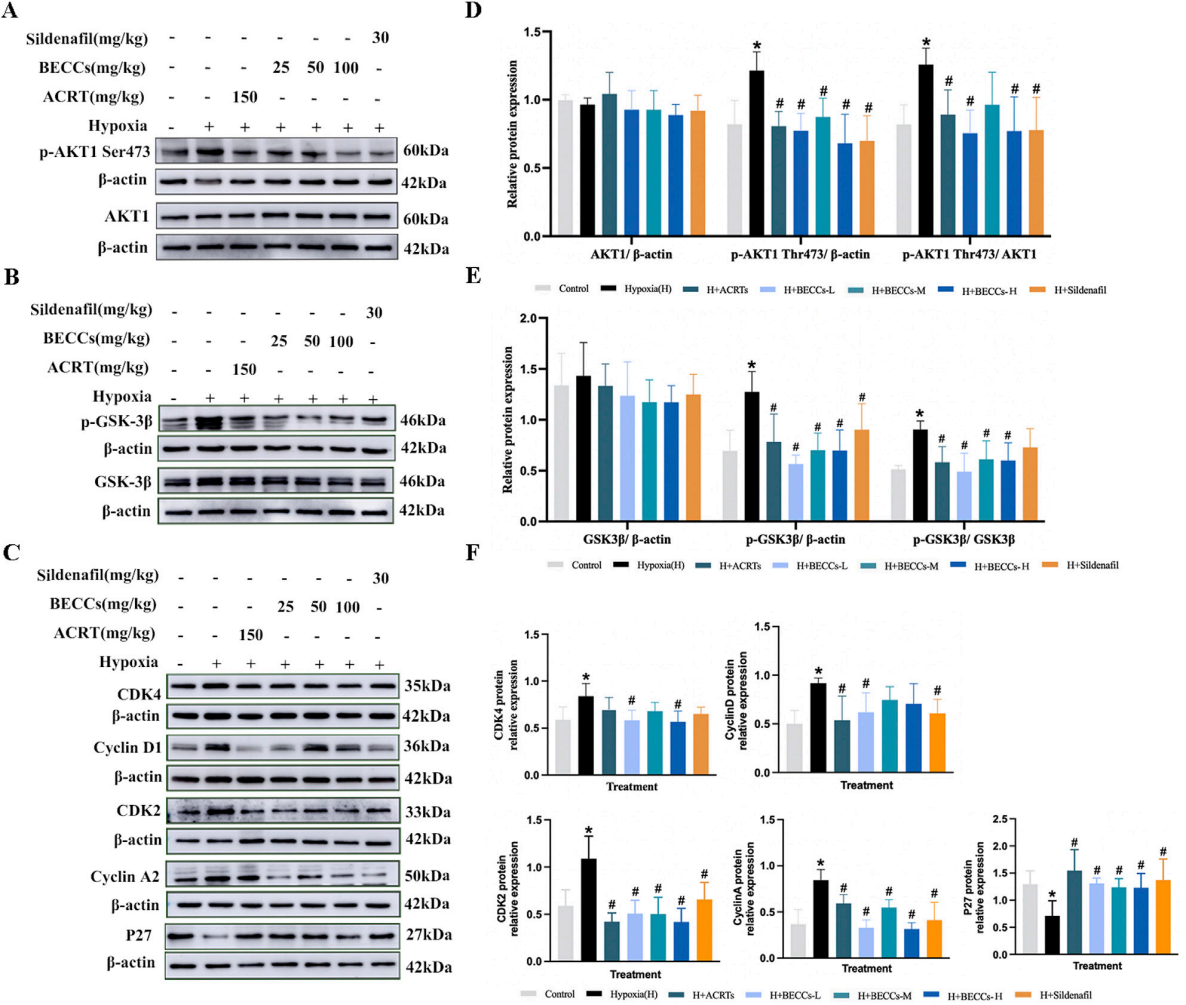
BECCs regulate the AKT/GSK3β/CDK/cyclin signaling pathway in HAPH rats. **(A–C)** Primitive bands of p-AKT1 S473, AKT1, p-GSK3β, GSK3β, CDK4, cyclin D1, CDK2, cyclin A, and P27 by Western blots in lung tissues. **(D–F)** Quantitative evaluation of p-AKT1 (S473), AKT1, p-GSK3β, GSK3β, CDK4, cyclin D1, CDK2, cyclin A, and P27 in the lung tissues. n = 5. All data are represented as the mean ± SD. *p < 0.05 vs. control group and ^#^ *p* < 0.05 vs. hypoxia group.

Since AKT1 is one of the potential targets of ACRT in preventing HAPH, a Western blot assay was used to examine whether BECCs affected AKT1 levels in HAPH rat lung tissues. As shown in Figures 4A, D, the p-AKT1(S473) levels were significantly higher under hypoxia than those in the control group, which were attenuated in the presence of ACRT, BECCs, and sildenafil (*p* < 0.05, Figures 4A, D). Nonetheless, no notable variations were found in the total protein levels of AKT1 among different groups (*p* > 0.05, Figures 4A, D). Since glycogen synthase kinase 3β (GSK3β) is one of the downstream proteins of AKT1 associated with vascular remodeling, we next sought to detect the impact of BECCs on the GSK3β protein. Compared to normoxia, p-GSK3β expression levels were elevated under hypoxia but were downregulated by ACRT and BECCs. Sildenafil treatment only affected the p-GSK3β expression levels (*p* < 0.05, Figures 4B, E). However, the total GSK3β levels were not altered (*p* > 0.05, Figures 4B, E).

### BECC treatment downregulated the CDK/cyclin pathway in HAPH rats

We further explored the role of BECCs in the alterations of cell cycle regulators. Cyclin D1, cyclin-dependent kinase 4 (CDK4), cyclin A2, and CDK2 levels were significantly higher under hypoxia than those in the control group, accompanied by a lower level of p27 (a CDK negative regulatory protein). Treatment with BECCs partially downregulated the hypoxia-induced increases in cyclin D1 and CDK4 levels in HAPH rats. For CDK2, cyclin A2, and P27 protein levels, BECC treatment significantly reversed the expression changes induced by hypoxia (*p* < 0.05, Figures 4C, F). Interestingly, ACRT and sildenafil treatments did not affect the hypoxia-induced elevation of CDK4 protein levels in rat lung tissues (*p* > 0.05, Figures 4C, F).

### Effect of BECCs on upstream kinases of AKT in HAPH rats

**FIGURE 5:**
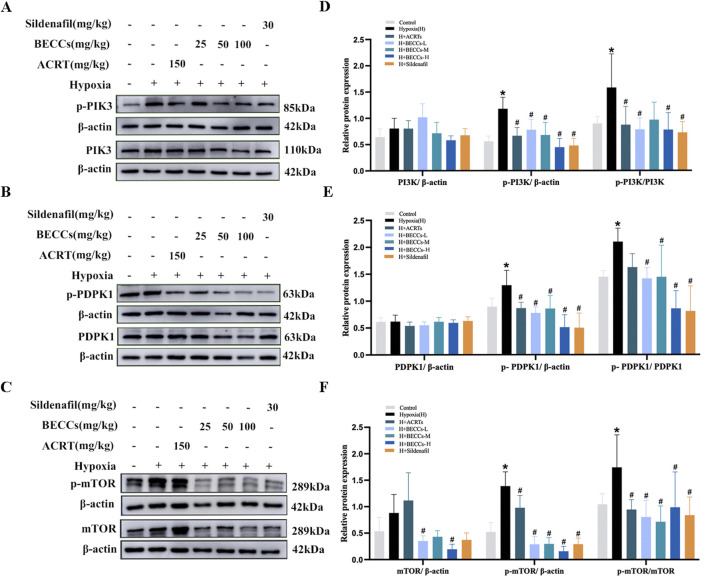
BECCs regulate PI3K, PDPK1, and mTOR protein levels in HAPH rats. **(A–C)** Primitive bands of p-PI3K, PI3K, p-PDPK1, PDPK1, p-mTOR, and mTOR by Western blots in lung tissues. **(D–F)** Quantitative evaluation of p-PI3K, PI3K, p-PDPK1, PDPK1, p-mTOR, and mTOR in lung tissues. n = 5. All data are represented as the mean ± SD. *p < 0.05 vs. control group and ^#^ *p* < 0.05 vs. hypoxia group.

To clarify whether BECCs directly act on AKT1 or interact with the upstream kinases of AKT1, including PI3K (Vanhaesebroeck et al., 2010), phosphoinositide-dependent protein kinase 1(PDPK1) (Calleja et al., 2009; Calleja et al., 2007), and mTORC2 (Liu et al., 2014a) proteins, we analyzed the impact of BECCs on these proteins in HAPH rats. The results showed that the phosphorylation levels of PI3K, PDPK1, and mTORC2 were all elevated in the hypoxia group compared to those in the control group, and treatment with BECCs, ACRT, and sildenafil markedly downregulated p-PI3K, p-PDPK1, and p-mTORC2 expression levels induced by hypoxia. The increased levels of total mTORC2 induced by hypoxia were decreased via BECC treatment (*p* < 0.05, Figures 5A–F), while PDPK1 and PI3K total levels did not alter notably (*p* > 0.05, Figures 5A–F).

### Evaluation of anti-proliferative combination from BECCs

**FIGURE 6:**
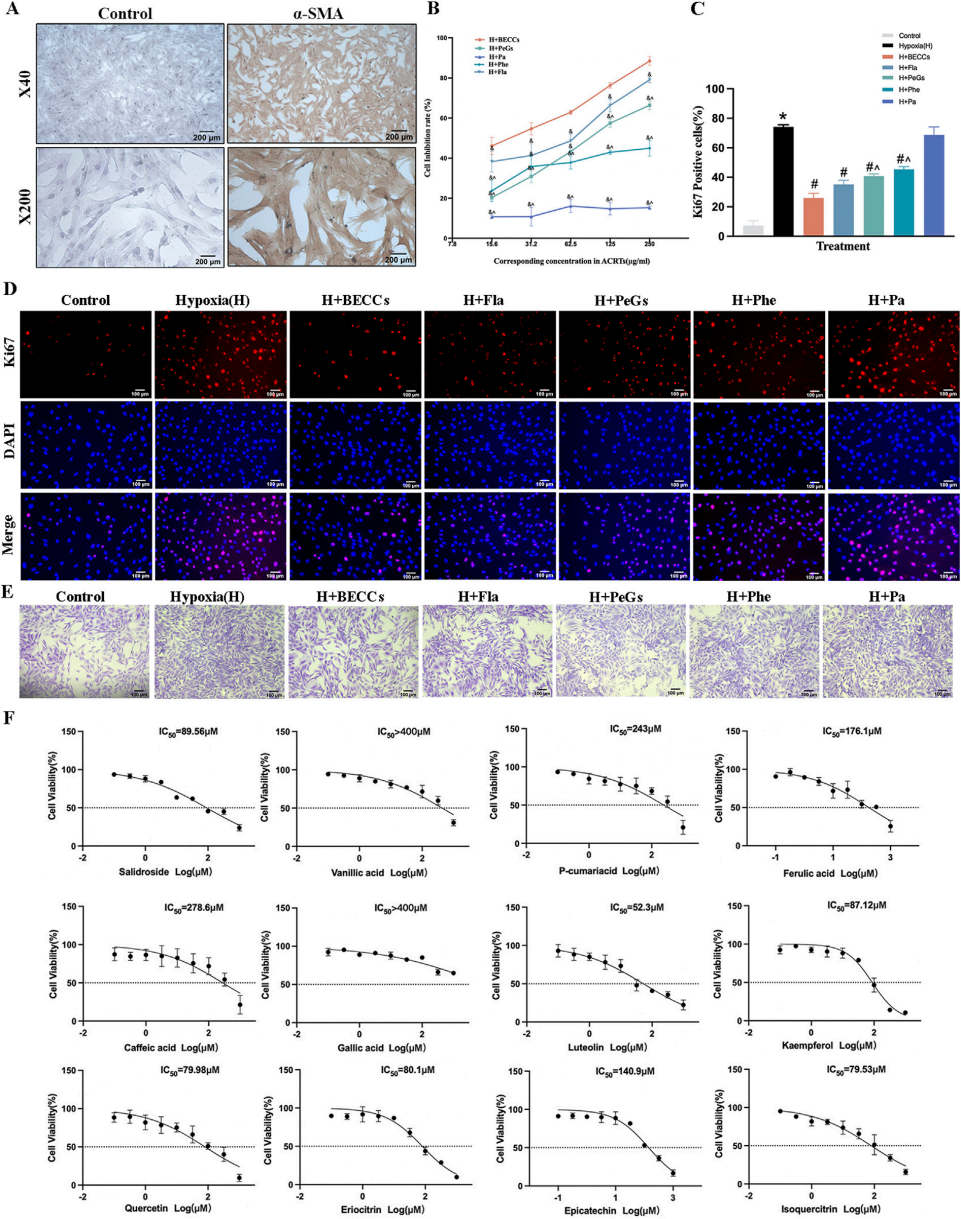
Anti-proliferative effect and dominant combination of BECCs in PASMCs under hypoxic conditions. FLAs, flavonoids; PeGs, phenylethanoid glycosides; PhEs, phenylpropanoids; PAs, phenolic acids. **(A)** Immunocytochemical analysis identified α-SMA-positive (brown) cells as PASMCs. **(B)** Dose-response curves of BECCs, FLAs, PeGs, PhEs, and PAs repress cell viability under 3% O_2_ for 24 h, n = 3. **(C, D)** Ki67 immunofluorescence and quantitative evaluation of BECCs in hypoxia-induced PASMCs assess cell proliferation (scale bar = 100 μm, n = 3). **(E)** Crystal violet assays in hypoxia-induced PASMCs (scale bar = 100 μm, n = 3). **(F)** Dose-response curves of 12 compounds in BECCs under 3% O_2_ for 24 h, n = 3. All data are represented as the mean ± SD. *p < 0.05 vs. control group, ^#^ *p* < 0.05 vs. 3% O_2_ group, ^&^ *p* < 0.05 vs. BECC group, and ^^^ *p* < 0.05 vs. FLA group.

The primary PASMCs isolated from SD rats were nearly all α-SMA-positive, as verified by immunohistochemical analysis (Figure 6A). Based on the chemical structures of the 12 ingredients in BECCs, they were categorized into four classes, namely, FLAs, PeGs, PhEs, and PAs. To further validate the anti-proliferative effects of BECCs on PASMCs and screen out the main group charged with the anti-proliferative effect, we prepared FLAs, PeGs, PhEs, PAs, and BECCs by combining relevant components following the respective content in BECCs (Supplementary Table S1). The IC_50_ value of each combination for PASMC proliferation under 3% O_2_ conditions was shown as follows: BECCs, IC_50_ = 29.47 μg/mL; FLAs, IC_50_ = 44.17 μg/mL; PeGs, IC_50_ = 90.91 μg/mL; PhEs, IC_50_ = 238.9 μg/mL; and PAs: IC_50_ > 400 μg/mL. All four groups showed inhibitory effects on hypoxia-induced PASMC proliferation, which exhibited a synergistic interaction, and among them, FLAs exhibited the strongest anti-proliferative effect (*p* < 0.05, Figure 6B). According to the IC_50_ value, 50 μg/mL of BECCs was selected for the subsequent cell experiments. Consistently, compound combinations of corresponding concentrations were prepared in 50 μg/mL of BECCs, including 18.0 μg/mL of FLAs, 14.3 μg/mL of PeGs, 2.2 μg/mL of PhEs, and 15.5 μg/mL of PAs.

We further evaluated the effects of combinatory compounds in inhibiting the proliferation of PASMCs under 3% O_2_ conditions via Ki67 immunofluorescence. The results showed that the proportion of Ki67-positive PASMCs was remarkably increased under hypoxia compared to that under normoxic conditions, which could be attenuated by treatment with BECCs (50 μg/mL), FLAs (18.0 μg/mL), PeGs (14.3 μg/mL), and PhEs (2.2 μg/mL), respectively (*p* < 0.05, Figures 6C, D). PAs (15.5 μg/mL) showed no significant inhibitory effect, while PeGs and PhEs demonstrated lower efficacy than FLAs (*p* < 0.05, Figures 6C, D). Crystal violet assays showed that cell viability was increased under hypoxia, which was decreased in the presence of BECCs and combinatory compounds (Figure 6E). Notably, FLAs might present evident inhibitory effects on hypoxia-induced PASMC proliferation. Therefore, we speculated that the combination of FLAs might be the dominant combination primarily responsible for the anti-proliferative activity. In addition, dose–effect curves of 12 single active compounds on hypoxia-induced proliferation and IC_50_ values are listed in Figure 6F; quercetin, luteolin, kaempferol, salidroside, eriocitrin, and isoquercitrin exhibited potent anti-proliferative activity with lower IC_50_ values compared to other compounds.

### Flavonoids inhibited the proliferation of PASMCs under hypoxia via the PI3K/AKT axis

**FIGURE 7:**
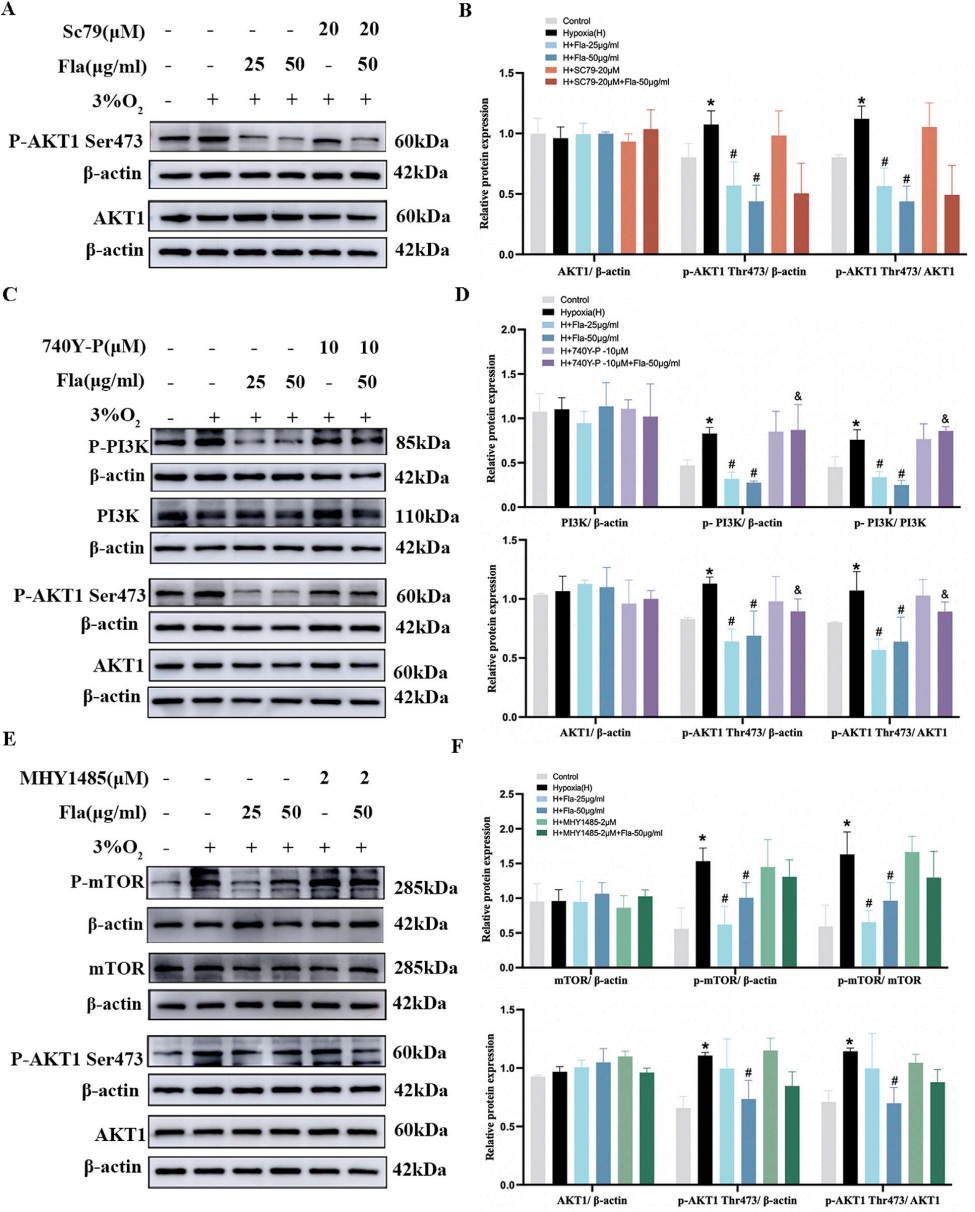
Flavonoids inhibit the proliferation of PASMCs under hypoxic conditions by inhibiting the PI3K/AKT axis. **(A, B)** Primitive bands and quantitative densities of p-AKT1 Ser473 and AKT1 with or without Sc79 (20 μM) by Western blots in PASMCs under 3% O_2_. **(C, D)** Primitive bands and quantitative densities of p-PI3K, PI3K, p-AKT1 Ser473, and AKT1 with or without 740Y-P (10 μM) by Western blots in PASMCs under 3% O_2_. **(E, F)** Primitive bands and quantitative densities of p-PDPK1, PDPK, p-AKT1 Ser473, and AKT1 with or without MYH1485 (2 μM) in PASMCs by Western blots under 3% O_2_. n = 3. All data are represented as the mean ± SD. *p < 0.05 vs. control group, ^#^ *p* < 0.05 vs. 3% O_2_ group, and *p* < 0.05 vs. 3% O_2_ + Fla-50 μg/ml group.

To further explore the mechanism of FLAs on PASMC proliferation, we detected the AKT1, PI3K, PDPK1, and mTOR protein expressions with corresponding pharmacological agonists. Based on IC_50_ values, FLA concentrations of 25 and 50 μg/mL were selected for subsequent experiments. Specifically, we treated hypoxia-induced PASMCs with Sc79 (AKT activator) and found no significant changes in total AKT1 protein expression among different groups (*p* > 0.05, Figures 7A, B). The phosphorylation of AKT1 (S473) was upregulated under hypoxia compared to that in the control group, which was attenuated by FLA treatment (*p* < 0.05, Figures 7A, B); however, co-treatment with Sc79 (20 μM) did not reverse FLA-induced (50 μg/mL) suppression of AKT1-S473 phosphorylation (*p* > 0.05, Figures 7A, B).

Then, we used 740 Y-P (PI3K activator) to ascertain the role of FLAs in PI3K protein regulation. No significant changes were observed in the total levels of PI3K and AKT1 proteins among all groups (*p* > 0.05, Figures 7C, D). The levels of p-AKT1 (S473) and p-PI3K were elevated under hypoxia compared to those in the normoxia group, which were downregulated by FLA treatment (*p* < 0.05, Figures 7C, D). Interestingly, the activation of the PI3K/AKT axis using 740 Y-P (10 μM) diminished the effect of FLA (50 μg/mL) on the PI3K/AKT pathway (*p* < 0.05, Figures 7C, D).

Meanwhile, 3% O_2_-stimulated PASMCs were treated with FLAs (25 and 50 μg/mL) and MHY1485 (2 μM, mTOR activator). The data showed that p-mTOR and p-AKT1 (S473) levels under hypoxia were significantly increased compared to those in the control group, and intervention with FLAs (50 μg/mL) markedly downregulated p-mTOR and p-AKT1 (S473) levels (*p* < 0.05, Figures 7E, F), without affecting their total protein levels (*p* > 0.05, Figures 7E, F). However, using MHY1485 (2 μM) did not significantly weaken the effect of FLAs (50 μg/mL) (*p* > 0.05, Figures 7E, F).

**FIGURE 8:**
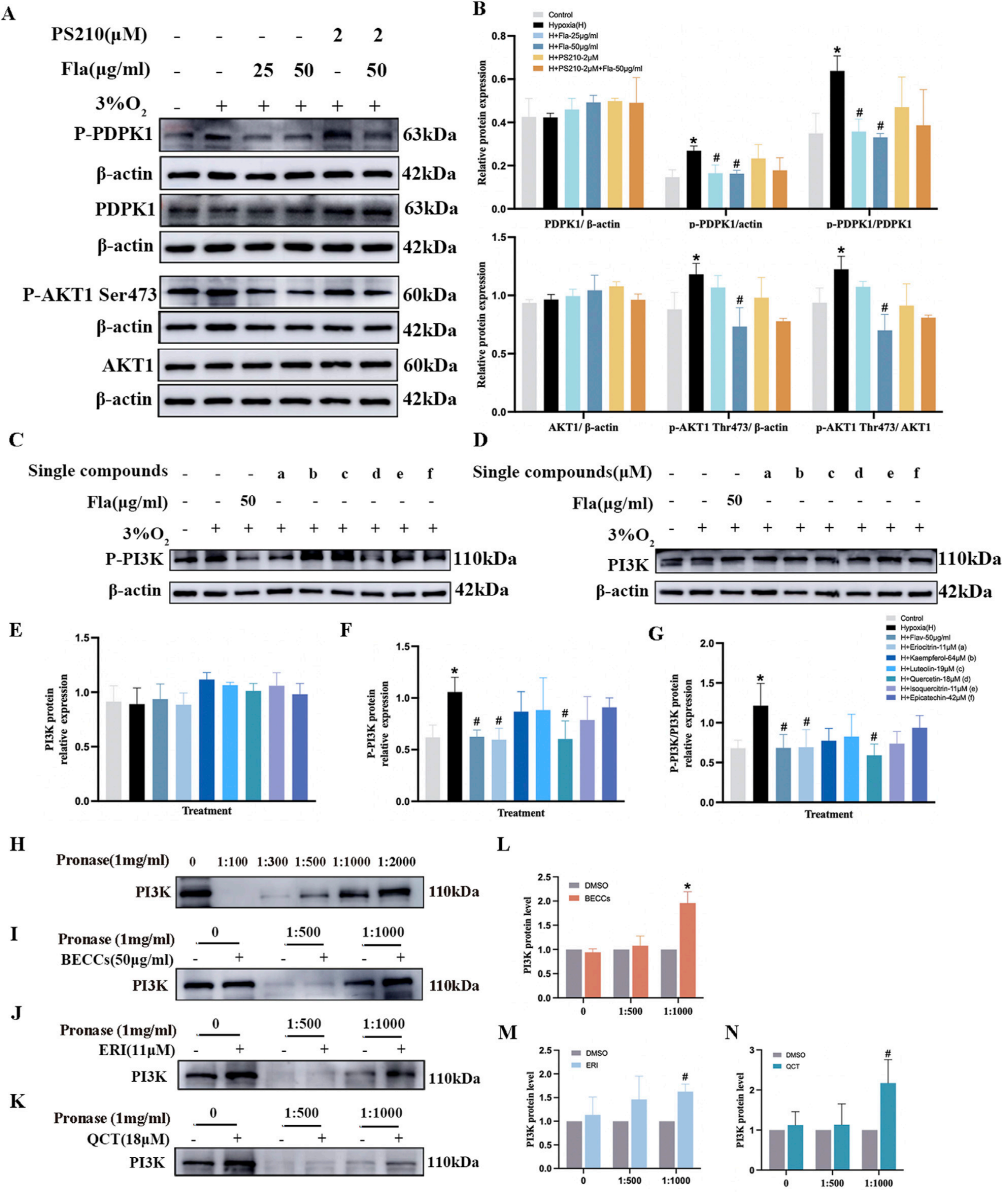
Eriocitrin and quercetin are responsible for anti-proliferation by targeting the PI3K protein in PASMCs under hypoxic conditions. ERI, eriocitrin; QCT, quercetin. **(A, B)** Primitive bands and quantitative evaluation of p-mTOR, mTOR, p-AKT1 (Ser473), and AKT1 with or without PS210 (2 μM) by Western blotting in PASMCs under 3% O_2_. n = 3. All data are represented as the mean ± SD. *p < 0.05 vs. control group, ^#^ *p* < 0.05 vs. 3% O_2_ group, and *p* < 0.05 vs. 3% O_2_ + FLA-50 μg/ml group. **(C–G)** Primitive bands and quantitative densities of p-PI3K and PI3K by Western blots. n = 3. All data are represented as the mean ± SD. *p < 0.05 vs. control group and ^#^ *p* < 0.05 vs. 3% O_2_ group. **(H–N)** BECC, ERI, and QCT treatment increased the stability of PI3K in PASMC protease lysates by the DARTS experiment. **(H–K)** Primitive Western blots of PI3K. **(L–N)** Quantitative evaluation of PI3K levels. n = 3. All data are represented as the mean ± SD. *p < 0.05 vs. DMSO group.

Additionally, we further explored the effect of Fla on the PDPK1 protein using PS210 (PDPK1 activator). Compared to the normoxia group, p-PDPK1 and p-AKT1 (S473) protein levels were prominently increased under hypoxia conditions. Treatment with 50 μg/mL of FLAs reduced the p-PDPK1 and p-AKT1 (S473) levels (*p* < 0.05, Figures 8A, B) without affecting PDPK1 and AKT1 total protein levels (*p* > 0.05, Figures 8A, B). Notwithstanding, between the 3% O_2_ + FLA 50 μg/mL + PS210 2 μM group and 3% O_2_ + FLA 50 μg/mL group, there was no noticeable alteration in p-PDPK1 and p-AKT1 (S473) levels (*p* > 0.05, Figures 8A, B).

Collectively, the abovementioned results revealed that FLAs inactivated the PI3K/AKT signaling pathway to exert the anti-proliferative effect on PASMCs under hypoxia.

### Eriocitrin and quercetin were screened as primary compounds suppressing PASMC proliferation via targeting PI3K under hypoxia

Subsequently, we observed the effects of six compounds in FLAs on PI3K expression in hypoxia-induced PASMCs. As shown in Figures 8C–G, compared to those in the normoxia group, p-PI3K protein expression levels were elevated in the 3% O_2_ group (*p* < 0.05). Treatment with FLAs (50 μg/mL), eriocitrin (ERI, 11 μM), and quercetin (QCT, 18 μM), the levels of p-PI3K and p-PI3K/PI3K were downregulated compared to those in the 3% O_2_ group (*p* < 0.05, Figures 8C–G). However, treatment with kaempferol (64 μM), luteolin (19 μM), isoquercitrin (11 μM), and epicatechin (42 μM), the expression of p-PI3K did not show significant changes compared to those in the hypoxia group (*p* > 0.05, Figures 8C–G). The levels of total PI3K protein did not show statistical differences among different groups (*p* > 0.05, Figures 8C–G). To further explore the binding affinity among ERI, QCT, and PI3K, the drug affinity responsive target stability (DARTS) assay was conducted and showed that when the protease-to-protein ratio was 1:500 and 1:1,000, there was still a certain amount of protein remaining, which was remarkably different from that in the control group; thus, these two ratios were selected for subsequent studies (Figure 8H). We observed that BECCs, together with PI3K, exhibited a binding affinity, as evidenced by the enhanced resistance of PI3K to proteolysis upon association with BECCs (Figures 8I, L). Meanwhile, ERI and QCT also partially suppressed the pronase digestion on PI3K, which confirmed that ERI and QCT were directly bound to PI3K (*p* < 0.05, Figures 8J–N). Moreover, the well-binding activities among ERI, QCT, and PI3K were demonstrated through molecular docking (Supplementary Figure S3). Based on the abovementioned findings, we screened ERI and QCT and selected the PI3K target for further anti-proliferative validation in hypoxia-stimulated PASMCs.

### Effects of eriocitrin or quercetin on anti-proliferation and antioxidation of PASMCs under hypoxia

**FIGURE 9:**
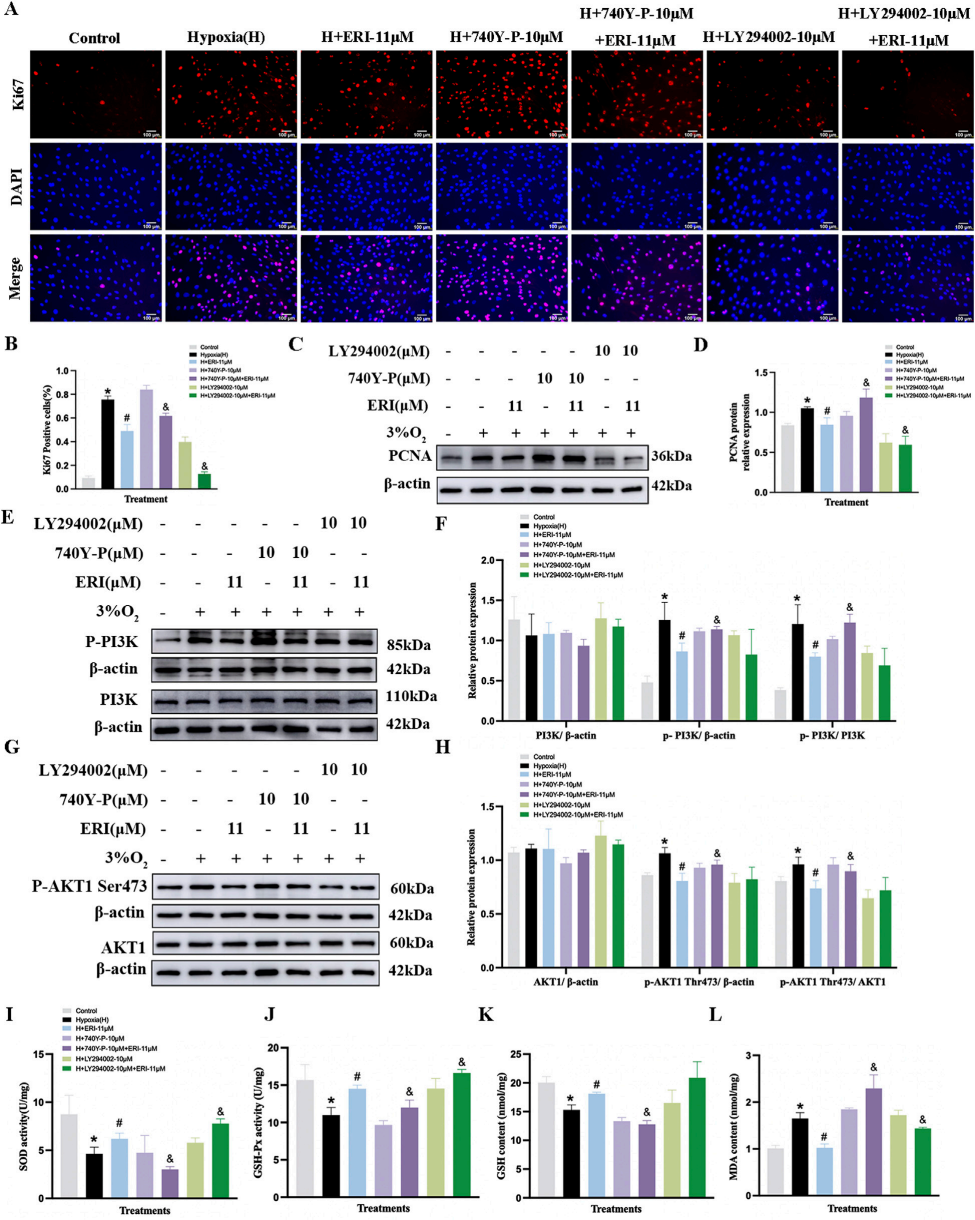
Treatment with eriocitrin in PASMC proliferation and antioxidation under hypoxic conditions. **(A, B)** Eriocitrin in cell proliferation were assessed using Ki67 immunofluorescence and quantitative evaluation in hypoxia-induced PASMCs (n = 3, scale bar = 100 μm). **(C–H)** Primitive Western blots and quantitative densities of PCNA, p-PI3K, PI3K, p-AKT1 (Ser473), AKT1 with or without 740Y-P (10 μM), LY294002 (10 μM), or eriocitrin (11 μM) in PASMCs under 3% O_2_ for 24 h **(I–L)** Quantitative evaluation of SOD and GSH-Px activities and GSH and MDA contents in 3% O_2_-induced PASMCs. n = 3. All data are represented as the mean ± SD. *p < 0.05 vs. control group, ^#^ *p* < 0.05 vs. 3% O_2_ group, and *p* < 0.05 vs. 3% O_2_ + ERI-11 μM.

We further validated the anti-proliferative efficacy of ERI and QCT on hypoxia-induced PASMCs. LY294002 (10 μM, PI3K antagonist) and 740Y-P (10 μM) were selected to determine whether ERI or QCT exerted the effect via targeting the PI3K protein. We found that the proportion of Ki67-positive PASMCs was elevated under 3% O_2_ conditions compared to that in the control group (Figures 9A, B, *p* < 0.05), and both ERI (11 μM) and QCT (18 μM) could significantly attenuate the changes (*p* < 0.05). However, ERI (11 μM)- or QCT (18 μM)-mediated effects on PASMC proliferation were abolished upon co-treatment with 740Y-P (10 μM). Additionally, ERI (11 μM) or QCT (18 μM) co-treatment with LY294002 (10 μM) could all effectively inhibit the increase in the positive expression of Ki67 in PASMCs induced by hypoxia (*p* < 0.05, Figures 9A, B).

**FIGURE 10:**
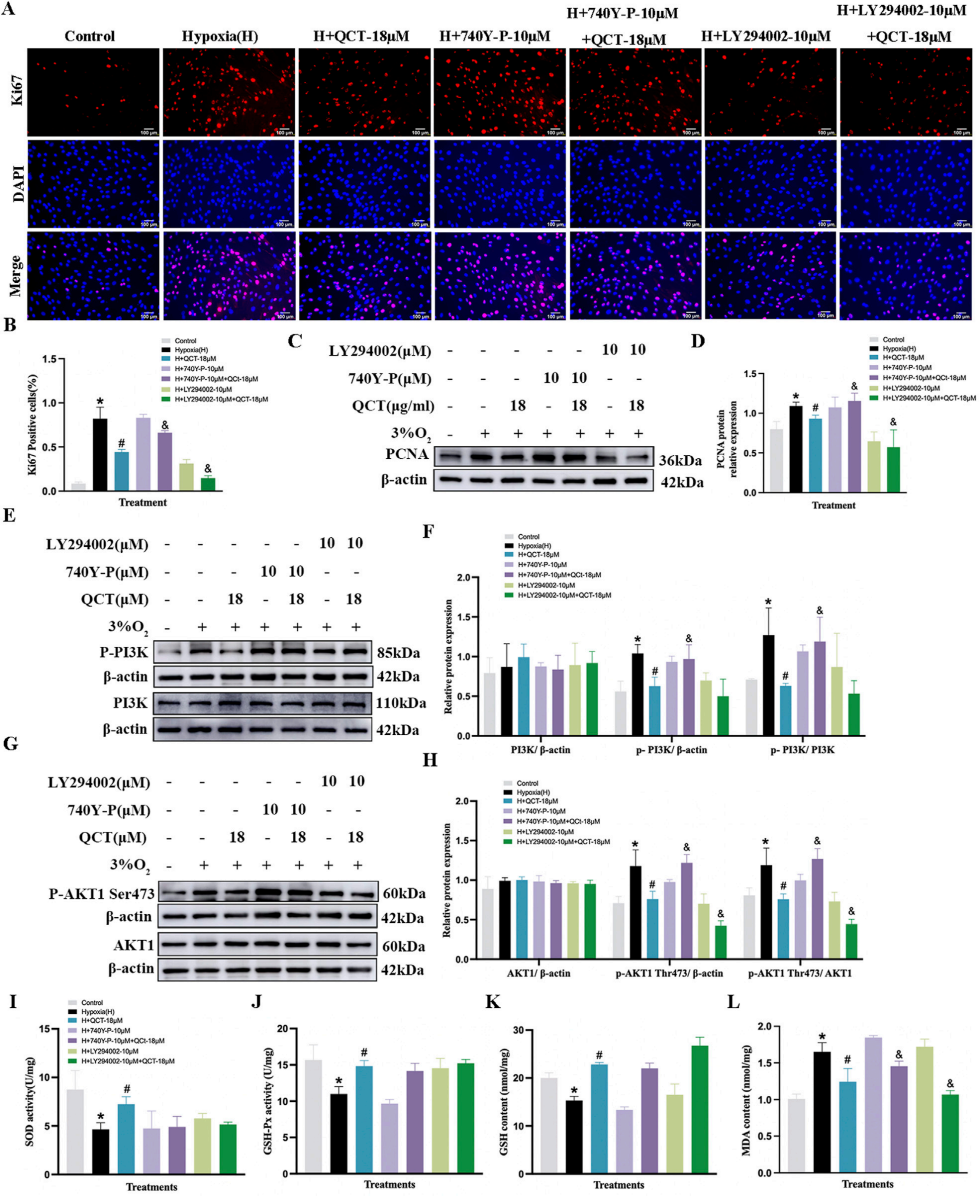
Treatment with quercetin in PASMC proliferation and antioxidation under hypoxia. **(A, B)** Quercetin in cell proliferation were assessed using Ki67 immunofluorescence and quantitative evaluation in hypoxia-induced PASMCs (n = 3, scale bar = 100 μm). **(C–H)** Primitive Western blots and quantitative densities of PCNA, p-PI3K, PI3K, p-AKT1 Ser473, AKT1 with or without 740Y-P(10 μM), LY294002(10 μM), or quercetin (18 μM) in PASMCs under 3% O_2_ for 24 h **(I–L)** Quantitative evaluation of SOD and GSH-Px activities and GSH and MDA contents in 3% O_2_-induced PASMCs. n = 3. All data represent mean ± SD. *p < 0.05 vs. control group, ^#^ *p* < 0.05 vs. 3% O_2_ group, and *p* < 0.05 vs. 3% O_2_ + QCT-18 μM.

Moreover, both ERI (11 μM) and QCT (18 μM) could inhibit the increased levels of PCNA, p-PI3K, and p-AKT1 (S473) in PASMCs under hypoxic conditions (*p* < 0.05, Figures 9C–H, 10C–H), while these inhibitory effects were eliminated by 740Y-P treatment (*p* < 0.05, Figures 9C–H, 10C–H). Meanwhile, when we co-treated hypoxia-induced PASMCs with LY294002, ERI (11 μM) did not enhance the effects of LY294002 on PI3K protein compared to ERI (11 μM) treatment alone, indicating that the primary signaling target of ERI was likely blocked (*p* > 0.05, Figures 9E, F). No significant changes were found in the total protein levels of PI3K and AKT1 across all groups (*p* > 0.05, Figures 9E–H, 10E–H).

Additionally, in the oxidant stress study, our results showed that 3% O_2_ exposure significantly decreased the levels of GSH, SOD, and GSH-Px, while it increased the content of MDA in PASMCs compared to that in the control group (*p* < 0.05, Figures 9I–L, 10I–L). Treatment with ERI (11 μM) and QCT (18 μM) increased the levels of SOD, GSH, and GSH-Px, while the MDA content was significantly reduced in PASMCs (*p* < 0.05, Figures 9I–L, 10I–L). Furthermore, ERI (11 μM)-mediated anti-oxidative effects on PASMCs were eliminated upon co-treatment with 740Y-P (10 μM), while co-treatment with LY294002 (10 μM) enhanced the effects. However, the anti-oxidative effects of QCT were neither abolished by co-treatment with 740Y-P (10 μM) nor enhanced by co-treatment with LY294002 (10 μM) (*p* < 0.05, Figures 9I–L, 10I–L).

## Discussion

The chemical constituents of herbs are diverse and complex, encompassing a wide range of structural types (Song et al., 2015). Accumulating studies have identified numerous bioactive small molecules from herbs. However, their holistic effects are attributed to combinatorial components rather than individual compounds (Liu et al., 2014b). Hence, based on our previous research that ACRT could alleviate HAPH (Nan et al., 2018), the present study mainly evaluated a combination of abundant compounds, known as BECCs, to determine whether it could represent the protective effect of ACRT against HAPH.

In this study, HAPH rat models were created in a hypobaric chamber, which simulated a hypoxic environment at a height of 5,000 meters. The results showed that the combination of 12 abundant compounds in ACRT not only suppressed mPAP and improved right ventricular hypertrophy but also significantly attenuated pulmonary vascular remodeling, explaining the majority of holistic effects of ACRT in HAPH rats. Hypoxia induces excessive proliferation of PASMCs, which, in turn, triggers pulmonary vascular remodeling (Liu et al., 2022). A previous study indicated that exposure to 3% O_2_ for 24 h could lead to hyper-proliferation of PASMCs (Zhang et al., 2023). PCNA as a key marker for evaluating cell proliferation is related to the synthesis level of cellular DNA (Strzalka and Ziemienowicz, 2011). Our results indicated that α-SMA and PCNA levels were significantly elevated in the pulmonary arteries of HAPH rats, while BECC treatment could significantly decrease the expressions. Western blot analyses further demonstrated that the BECCs decreased PCNA expression in the lung tissue. These findings suggested that pulmonary vascular remodeling under hypoxia conditions could be attenuated by BECCs through the suppression of PASMC proliferation. Therefore, the BECCs were as effective as ACRT on pulmonary artery vascular remodeling in HAPH rats. To further confirm our findings, we performed Ki67 immunofluorescence and crystal violet assays, both of which confirmed that BECCs lowered hypoxia-induced PASMC proliferation.

Moreover, SOD, a biomarker of oxidative stress in PH, protects cells against harm caused by superoxide radicals. Restoring SOD activity can alleviate pulmonary artery remodeling (Wang et al., 2022a). Furthermore, PH leads to increased lipid peroxidation, manifested as an increase in MDA levels (Cracowski et al., 2001). GSH-Px catalyzes the reaction between GSH and hydrogen peroxide to produce GSSG and H_2_O, thereby inhibiting ROS-mediated damage (Pena et al., 2024). Our results showed that both the decreased levels of SOD, GSH-Px, and GSH and the increased level of MDA caused by hypoxia in the lung tissue were effectively reversed by the treatment of BECCs and ACRT, which proved that BECCs alleviated the oxidative stress response.

The abovementioned data validate that BECCs and ACRT exert comparable effects in ameliorating pulmonary vascular remodeling in HAPH rats, thereby supporting the rationale for designating this set of 12 compounds as the BECCs of ACRT. We next preliminarily explored the mechanism of BECCs on pulmonary vascular remodeling in HAPH. Protein phosphorylation, a popular and vital post-translational modification, widely affects protein activity and functions. Many phosphorylated proteins are all linked to the progression of PH. In our phosphoproteomic analysis, we observed that AKT1 was one of the major potential targets. As one of the dominant proteins in the pathogenesis of HAPH, AKT1 was involved in both cellular proliferation and apoptosis. Studies have found that pulmonary vascular remodeling was reversed, and PAH development was markedly attenuated by targeting AKT1 (Yang et al., 2021; Tang et al., 2024) or knocking out the *AKT1* gene (Zuo et al., 2021). Furthermore, mechanistic studies revealed that the components of BECCs inhibit PASMC proliferation by targeting AKT protein activity. Specifically, luteolin can improve PH through the PI3K/AKT pathway in rats (Ji et al., 2022). Kaempferol attenuates pulmonary vascular remodeling in HAPH rats by inhibiting PASMC proliferation via the AKT/GSK3β/cyclin signaling pathway (Zhang et al., 2023). Isoquercetin prevents PASMC proliferation by blocking PDGF-Rβ signaling and its downstream AKT/GSK3β and ERK1/2 pathways (Zhang et al., 2017). Quercetin has been investigated for its therapeutic potential in PAH through the modulation of the AKT pathway (Ungurianu et al., 2024). Hence, based on previous reports and our phosphoproteomic analysis, we speculate that BECCs alleviate HAPH by regulating AKT1 and AKT1-mediated signaling pathways. Furthermore, the PI3K/AKT signaling pathway plays a vital role under hypoxic conditions (Xue et al., 2023; Hu et al., 2021). Therefore, to ascertain whether BECCs directly inhibit the AKT1 activity or act on the upstreaming proteins, we tested the protein levels and found that BECCs significantly decreased the hypoxia-induced phosphorylation level of AKT1 and the upstream kinases, including PI3K, PDK1, and mTORC2 *in vivo*. Interestingly, our results showed that BECCs affected all four proteins, aligning with the multiple-component and multiple-target features associated with natural products.

We further clarified the AKT1 downstream proteins correlated with the pathogenesis of HAPH. Previous studies have shown that the AKT/GSK3β pathway is implicated in the proliferation and anti-apoptosis of PASMCs under hypoxic conditions (Gu et al., 2023; Zhang et al., 2023). The downregulation of p-AKT and p-GSK3β activity can alleviate PH in rats (Nie et al., 2023). Our findings were consistent with these studies, indicating that the phosphorylation level of GSK3β was substantially elevated in the hypoxia group; however, this effect was reversed following BECC administration. In addition, researchers have proved that PI3K/AKT is also associated with CDKs and cyclin regulation proteins, which are critically important for cell cycle progression and proliferation (Hou et al., 2020). Cyclin proteins activate CDKs in the G1 phase and induce cells to transition from the G1 to S phase (Hume et al., 2020). Moreover, it has been confirmed that the pharmacodynamic effect of ACRT on HAPH is based on downregulation of CDK4 and cyclin D expression levels (Nan et al., 2018). In our study, BECCs decreased cyclin D1-CDK4 and cyclin A-CDK2 protein expressions in the lung tissue of HAPH rats. Additionally, P27kip1 protein, a key negative regulator of the cell cycle that inhibits the G1-to-S transition and suppresses cell proliferation, was upregulated after treatment with BECCs (Ray et al., 2009). Collectively, these results demonstrated that the pharmacodynamic effects of BECCs in HAPH are associated with the AKT signaling pathway.

Since BECCs have been validated as biologically equivalent to ACRT in terms of anti-proliferative activity in both HAPH rats and hypoxia-stimulated PASMC models, evaluating the combinatory components within these 12 ingredients with antiproliferative properties is feasible. In this study, we used a chemical family-based approach to comprehensively explore multicomponent interactions in BECCs. The term “chemical family” is defined as a set of ingredients with similar structures and pharmacophores, which may have similar pharmacological properties (Song et al., 2016). The compounds identified in the herb can generally be classified into multiple chemical categories based on similar pharmacophores and pharmacological properties, albeit with varying contents (Long et al., 2015). Accordingly, BECCs were divided into FLA, PeG, PhE, and PA groups to determine the dominant component in charge of the anti-proliferative effects. We observed that FLAs exerted strong anti-proliferative effects, nearly comparable to the whole extract of BECCs. This revealed that FLAs were the predominant contributors to inhibiting the proliferation of PASMCs. Several studies have presented consistent evidence confirming the therapeutic potential of FLAs against HAPH, attributing this effect to their notable anti-proliferative activity (Wang et al., 2021; Zhang et al., 2024a; Shen et al., 2022). In our study, we observed that PAs did not exhibit evident inhibitory effects on hypoxia-induced PASMC proliferation. We considered that the effect of PAs on arterial remodeling in HAPH may not be directly related to PASMC proliferation, but rather to other mechanisms, such as migration, phenotype changes, decreased apoptosis of PASMCs, or endothelial dysfunction. Vanillic acid and gallic acid, two components of PAs in our study, exhibit inhibitory activity against arginase (Wang et al., 2022a, Wang et al., 2022b). Since arginase plays a crucial role in the development of PH, its deletion in pulmonary endothelial cells attenuates PH by increasing NO levels, thereby reducing pulmonary resistance and alleviating PH (Cowburn et al., 2016; Chu et al., 2016). In addition, previous studies demonstrated that salidroside protects pulmonary artery endothelial cells against hypoxia-induced apoptosis via the AhR/NF-κB and Nrf2/HO-1 pathways (Lei et al., 2024); this may be the reason that the anti-proliferative effect of salidroside on hypoxia-induced PASMCs was not as significant as FLA combinations in our study. Further studies are essential to elucidate the underlying mechanisms.

Moreover, we explored the underlying mechanisms of FLAs on PASMC proliferation under hypoxic conditions. Our data showed that p-AKT1 levels increased under hypoxia, which decreased following FLA treatment. To understand whether FLAs repress hypoxia-induced PASMC proliferation by suppressing AKT activity or AKT-related upstream regulators, the AKT activator (Sc79), PI3K activator (740Y-P), mTOR activator (MHY1485), and PDPK1 activator (PS210) were used. Notably, we found that the FLA (50 μg/mL) mediated-effect on PASMC proliferation was eliminated when co-treated with the PI3K activator (740Y-P, 10 μM). Meanwhile, when the FLA (50 μg/mL) was combined with Sc79, MHY1485, and PS210, no statistical significance was observed compared to that in the FLA-alone group (50 μg/mL). It was considered that FLAs inhibited PASMC proliferation by specifically targeting the PI3K protein.

Based on the abovementioned findings, we further evaluated the suppressive effects of six compounds in the FLA family, including kaempferol, eriocitrin, quercetin, luteolin, isoquercitrin, and epicatechin, on PI3K activity. Interestingly, we found that only eriocitrin (11 μM) and quercetin (18 μM) decreased p-PI3K levels in PASMCs under hypoxia. These differences might be attributed to variations in experimental models, drug concentrations, cell type specificity, or distinct mechanisms of action. Specifically, studies have demonstrated that luteolin could ameliorate PH by protecting pulmonary vascular endothelial function via regulating the HIF-2α–Arg–NO axis and the PI3K–AKT–eNOS–NO signaling pathway (Ji et al., 2022). Luteolin could also inhibit the proliferation and migration of PASMCs induced by platelet-derived growth factor-BB (PDGF-BB) in a dose-dependent manner (Zuo et al., 2021). Quercetin was explored for its therapeutic potential in PH by inhibiting endothelial transdifferentiation (Huang et al., 2017). Isoquercitrin and kaempferol attenuated pulmonary vascular remodeling by inhibiting PASMC proliferation (Zhang et al., 2017; Zhang et al., 2023); however, the effective concentrations reported in these studies differed from those in our study. Further analysis is critical to illuminate the primary cause. In addition, PI3K agonists (740Y-P) and PI3K antagonists (LY294002) were used. The data revealed that eriocitrin (11 μM) and quercetin (18 μM) effectively inhibited PASMC proliferation by suppressing both PI3K and AKT1 activities. DARTS is a reliable method for determining binding affinity between target proteins and small molecules (Ren et al., 2021). To determine the interaction between PI3K and eriocitrin or quercetin, we performed a DARTS experiment. The results demonstrated a binding affinity between PI3K and BECCs, as well as between PI3K and eriocitrin or quercetin in PASMCs, which indicated that the anti-proliferative effects of eriocitrin and quercetin on hypoxia-induced PASMCs are associated with PI3K. The conclusion was also confirmed by molecular docking analysis. In addition, eriocitrin (11 μM) and quercetin (18 μM) also suppressed the hypoxia-induced oxidant stress in PASMCs. We further detected their antioxidation after the addition of PI3K agonists (740Y-P) and PI3K antagonists (LY294002). Our results showed that 740Y-P offset the effect of ERI (11 μM) on oxidative stress, whereas LY294002 enhanced it, which suggested that ERI reduced the damage of oxidative stress by targeting the PI3K protein at the cellular level. However, there was no significant difference between the administration of 3% O_2_ + QCT (18 μM) and 3% O_2_ + QCT (18 μM) + 740Y-P (10 μM)/LY294002 (10 μM). We considered that the antioxidation of QCT in hypoxia-induced PASMCs might be regulated by other pathways. Previous studies have shown that QCT attenuates oxidative stress in other diseases by modulating key signaling pathways such as SIRT1/AMPK (Feng et al., 2019; Shao et al., 2019), Nrf2-ARE (Costa et al., 2016), and ERK (Zhi et al., 2016). However, its antioxidant effects in HAPH remain largely unexplored. Further research is needed to identify the mechanisms.

**FIGURE 11:**
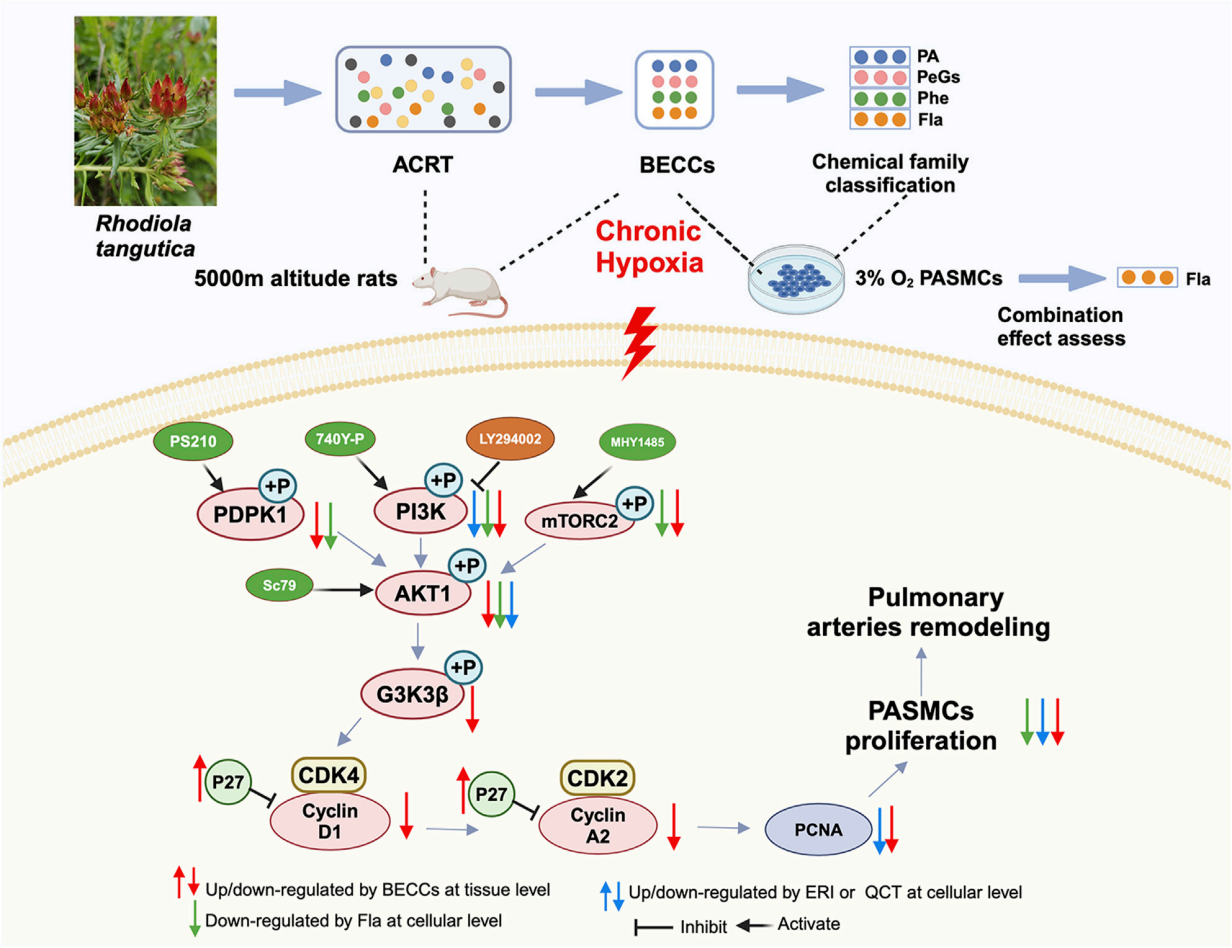
A sketch map shows that molecular mechanisms of BECCs suppress PASMC proliferation under hypoxic conditions by downregulating the PI3K/AKT pathway.

In short, our results suggested that BECCs could attenuate pulmonary vascular remodeling in HAPH rats by inhibiting PASMC proliferation. This protective effect is achieved through the downregulation of key proliferative regulators, including AKT, GSK3β, PCNA, cyclin D1, CDK4, cyclin A2, and CDK2, as well as the inhibition of p27Kip1 degradation. Particularly, FLA combinations in BECCs exhibited stronger anti-proliferative activity than others, acting as the dominant contributors by regulating PI3K rather than PDPK or mTOR pathways to inhibit AKT phosphorylation (Figure 11).

There are several limitations that should be noted. First, other than the anti-proliferative effect, BECCs probably possess many other pharmacological activities, such as protecting pulmonary vascular endothelial function, promoting PASMC apoptosis, anti-oxidation, and anti-inflammation; particularly, oxidative mechanisms in the pathogenesis of pulmonary hypertension are closely related to inflammation. However, inflammation was not examined in the present study; therefore, in the future study, all these effects should be further explored. Second, the ADME (absorption, distribution, metabolism, and excretion) processes of these structurally distinct compounds may exhibit differences *in vivo*. Given the complexity of biological systems, we will further investigate this through pharmacokinetic analysis. Third, the mechanism of BECCs targeting PI3K/AKT was not confirmed by gene knockout assays *in vitro*. Further in-depth research is currently underway.

## Conclusion

In this study, we detected a set of 12 compounds as BECCs of ACRT that significantly alleviated the progression of HAPH, reversed pulmonary artery remodeling in HAPH rats, and inhibited PASMC proliferation under hypoxia by targeting the PI3K–AKT signaling pathway. Moreover, based on chemical family classification, FLAs, particularly eriodictyol and quercetin, were identified as dominant contributors responsible for anti-proliferative activity through the regulation of PI3K rather than PDPK or mTOR pathways to inhibit AKT phosphorylation. These findings could be extended to improve quality control and clarify bioactive components of *R. tangutica* while also inspiring the exploration of combinatorial therapeutics for the treatment of HAPH.

## Data Availability

The original contributions presented in the study are included in the article/Supplementary Material; further inquiries can be directed to the corresponding author.

## References

[B1] Boezio B. Audouze K. Ducrot P. Taboureau O. (2017). Network-based approaches in pharmacology. Mol. Inf. 36 (10), 1700048. 10.1002/minf.201700048 28692140

[B2] Calleja V. Alcor D. Laguerre M. Park J. Vojnovic B. Hemmings B. A. (2007). Intramolecular and intermolecular interactions of protein kinase B define its activation *in vivo* . PLoS Biol. 5 (4), e95. 10.1371/journal.pbio.0050095 17407381 PMC1845162

[B3] Calleja V. Laguerre M. Parker P. J. Larijani B. (2009). Role of a novel PH-kinase domain interface in PKB/Akt regulation: structural mechanism for allosteric inhibition. PLoS Biol. 7 (1), e17. 10.1371/journal.pbio.1000017 19166270 PMC2628406

[B4] Chen T. Su S. Yang Z. Zhang D. Li Z. Lu D. (2022). Srolo Bzhtang reduces inflammation and vascular remodeling via suppression of the MAPK/NF-κB signaling pathway in rats with pulmonary arterial hypertension. J. Ethnopharmacol. 297, 115572. 10.1016/j.jep.2022.115572 35872290

[B5] Chen Y. Chen D. Liu S. Yuan T. Guo J. Fang L. (2019). Systematic elucidation of the mechanism of genistein against pulmonary hypertension via network pharmacology approach. Int. J. Mol. Sci. 20 (22), 5569. 10.3390/ijms20225569 31703458 PMC6888439

[B6] Christou H. Khalil R. A. (2022). Mechanisms of pulmonary vascular dysfunction in pulmonary hypertension and implications for novel therapies. Am. J. Physiol. Heart Circ. Physiol. 322 (5), H702–h724. 10.1152/ajpheart.00021.2022 35213243 PMC8977136

[B7] Chu Y. XiangLi X. Niu H. Wang H. Jia P. Gong W. (2016). Arginase inhibitor attenuates pulmonary artery hypertension induced by hypoxia. Mol. Cell Biochem. 412 (1-2), 91–99. 10.1007/s11010-015-2611-z 26608181

[B8] Costa L. G. Garrick J. M. Roquè P. J. Pellacani C. (2016). Mechanisms of neuroprotection by quercetin: counteracting oxidative stress and more. Oxid. Med. Cell Longev. 2016, 2986796. 10.1155/2016/2986796 26904161 PMC4745323

[B9] Cowburn A. S. Crosby A. Macias D. Branco C. Colaço R. D. Southwood M. (2016). HIF2α-arginase axis is essential for the development of pulmonary hypertension. Proc. Natl. Acad. Sci. U. S. A. 113 (31), 8801–8806. 10.1073/pnas.1602978113 27432976 PMC4978263

[B10] Cracowski J. L. Cracowski C. Bessard G. Pepin J. L. Bessard J. Schwebel C. (2001). Increased lipid peroxidation in patients with pulmonary hypertension. Am. J. Respir. Crit. Care Med. 164 (6), 1038–1042. 10.1164/ajrccm.164.6.2104033 11587993

[B11] Feng K. Chen Z. Pengcheng L. Zhang S. Wang X. (2019). Quercetin attenuates oxidative stress-induced apoptosis via SIRT1/AMPK-mediated inhibition of ER stress in rat chondrocytes and prevents the progression of osteoarthritis in a rat model. J. Cell Physiol. 234 (10), 18192–18205. 10.1002/jcp.28452 30854676

[B12] Gao R. J. Aikeremu N. Cao N. Chen C. Ma K. T. Li L. (2024). Quercetin regulates pulmonary vascular remodeling in pulmonary hypertension by downregulating TGF-β1-Smad2/3 pathway. BMC Cardiovasc Disord. 24 (1), 535. 10.1186/s12872-024-04192-4 39367342 PMC11451247

[B13] Gu C. Yang Z. Su S. Ma K. Nan X. Li Z. (2023). 4-Terpineol attenuates pulmonary vascular remodeling via suppressing PI3K/Akt signaling pathway in hypoxia-induced pulmonary hypertension rats. Toxicol. Appl. Pharmacol. 473, 116596. 10.1016/j.taap.2023.116596 37328117

[B14] He Y. Cao X. Liu X. Li X. Xu Y. Liu J. (2015). Quercetin reverses experimental pulmonary arterial hypertension by modulating the TrkA pathway. Exp. Cell Res. 339 (1), 122–134. 10.1016/j.yexcr.2015.10.013 26476374

[B15] Hou B. Li W. Li J. Ma J. Xia P. Liu Z. (2020). Tumor suppressor LHPP regulates the proliferation of colorectal cancer cells via the PI3K/AKT pathway. Oncol. Rep. 43 (2), 536–548. 10.3892/or.2019.7442 31894339 PMC6967159

[B16] Hu Z. Song Q. Ma H. Guo Y. Zhang T. Xie H. (2021). TRIM32 inhibits the proliferation and migration of pulmonary artery smooth muscle cells through the inactivation of PI3K/Akt pathway in pulmonary arterial hypertension. J. Bioenerg. Biomembr. 53 (3), 309–320. 10.1007/s10863-021-09880-w 33694017

[B17] Huang S. Zhu X. Huang W. He Y. Pang L. Lan X. (2017). Quercetin inhibits pulmonary arterial endothelial cell transdifferentiation possibly by akt and erk1/2 pathways. Biomed. Res. Int. 2017, 6147294. 10.1155/2017/6147294 28428963 PMC5385898

[B18] Hume S. Dianov G. L. Ramadan K. (2020). A unified model for the G1/S cell cycle transition. Nucleic Acids Res. 48 (22), 12483–12501. 10.1093/nar/gkaa1002 33166394 PMC7736809

[B19] Ji L. Su S. Xin M. Zhang Z. Nan X. Li Z. (2022). Luteolin ameliorates hypoxia-induced pulmonary hypertension via regulating HIF-2α-Arg-NO axis and PI3K-AKT-eNOS-NO signaling pathway. Phytomedicine 104, 154329. 10.1016/j.phymed.2022.154329 35843187

[B20] Lei W. Chen M. H. Huang Z. F. Chen X. Y. Wang J. X. Zheng J. (2024). Salidroside protects pulmonary artery endothelial cells against hypoxia-induced apoptosis via the AhR/NF-κB and Nrf2/HO-1 pathways. Phytomedicine 128, 155376. 10.1016/j.phymed.2024.155376 38503152

[B21] León-Velarde F. Maggiorini M. Reeves J. T. Aldashev A. Asmus I. Bernardi L. (2005). Consensus statement on chronic and subacute high altitude diseases. High. Alt. Med. Biol. 6 (2), 147–157. 10.1089/ham.2005.6.147 16060849

[B22] Li N. Su S. Xie X. Yang Z. Li Z. Lu D. (2024). Tsantan Sumtang, a traditional Tibetan medicine, protects pulmonary vascular endothelial function of hypoxia-induced pulmonary hypertension rats through AKT/eNOS signaling pathway. J. Ethnopharmacol. 320, 117436. 10.1016/j.jep.2023.117436 37979813

[B23] Liu P. Begley M. Michowski W. Inuzuka H. Ginzberg M. Gao D. (2014a). Cell-cycle-regulated activation of Akt kinase by phosphorylation at its carboxyl terminus. Nature 508 (7497), 541–545. 10.1038/nature13079 24670654 PMC4076493

[B24] Liu P. Yang H. Long F. Hao H. P. Xu X. Liu Y. (2014b). Bioactive equivalence of combinatorial components identified in screening of an herbal medicine. Pharm. Res. 31 (7), 1788–1800. 10.1007/s11095-013-1283-1 24549817 PMC4062815

[B25] Liu R. Xu C. Zhang W. Cao Y. Ye J. Li B. (2022). FUNDC1-mediated mitophagy and HIF1α activation drives pulmonary hypertension during hypoxia. Cell Death Dis. 13 (7), 634. 10.1038/s41419-022-05091-2 35864106 PMC9304375

[B26] Liu Z. Q. Yao G. L. Zhai J. M. Hu D. W. Fan Y. G. (2021). Kaempferol suppresses proliferation and induces apoptosis and DNA damage in human gallbladder cancer cells through the CDK4/CDK6/cyclin D1 pathway. Eur. Rev. Med. Pharmacol. Sci. 25 (3), 1311–1321. 10.26355/eurrev_202102_24836 33629301

[B27] Long F. Yang H. Xu Y. Hao H. Li P. (2015). A strategy for the identification of combinatorial bioactive compounds contributing to the holistic effect of herbal medicines. Sci. Rep. 5, 12361. 10.1038/srep12361 26198093 PMC4510521

[B28] Morales-Cano D. Menendez C. Moreno E. Moral-Sanz J. Barreira B. Galindo P. (2014). The flavonoid quercetin reverses pulmonary hypertension in rats. PLoS One 9 (12), e114492. 10.1371/journal.pone.0114492 25460361 PMC4252144

[B29] Nan X. Su S. Ma K. Ma X. Wang X. Zhaxi D. (2018). Bioactive fraction of Rhodiola algida against chronic hypoxia-induced pulmonary arterial hypertension and its anti-proliferation mechanism in rats. J. Ethnopharmacol. 216, 175–183. 10.1016/j.jep.2018.01.010 29325918

[B30] Nie X. Wu Z. Shang J. Zhu L. Liu Y. Qi Y. (2023). Curcumol suppresses endothelial-to-mesenchymal transition via inhibiting the AKT/GSK3β signaling pathway and alleviates pulmonary arterial hypertension in rats. Eur. J. Pharmacol. 943, 175546. 10.1016/j.ejphar.2023.175546 36706802

[B31] Pena E. El Alam S. Gonzalez C. Cortés I. Aguilera D. Flores K. (2024). Astaxanthin supplementation effects in right ventricle of rats exposed to chronic intermittent hypobaric hypoxia. Antioxidants (Basel) 13 (10), 1269. 10.3390/antiox13101269 39456521 PMC11504862

[B32] Polumackanycz M. Konieczynski P. Orhan I. E. Abaci N. Viapiana A. (2022). Chemical composition, antioxidant and anti-enzymatic activity of golden root (Rhodiola rosea L.) commercial samples. Antioxidants (Basel) 11 (5), 919. 10.3390/antiox11050919 35624783 PMC9137987

[B33] Poyatos P. Gratacós M. Samuel K. Orriols R. Tura-Ceide O. (2023). Oxidative stress and antioxidant therapy in pulmonary hypertension. Antioxidants (Basel) 12 (5), 1006. 10.3390/antiox12051006 37237872 PMC10215203

[B34] Ray A. James M. K. Larochelle S. Fisher R. P. Blain S. W. (2009). p27Kip1 inhibits cyclin D-cyclin-dependent kinase 4 by two independent modes. Mol. Cell Biol. 29 (4), 986–999. 10.1128/mcb.00898-08 19075005 PMC2643810

[B35] Ren Y. S. Li H. L. Piao X. H. Yang Z. Y. Wang S. M. Ge Y. W. (2021). Drug affinity responsive target stability (DARTS) accelerated small molecules target discovery: principles and application. Biochem. Pharmacol. 194, 114798. 10.1016/j.bcp.2021.114798 34678227

[B36] Shao Y. Yu H. Yang Y. Li M. Hang L. Xu X. (2019). A solid dispersion of quercetin shows enhanced Nrf2 activation and protective effects against oxidative injury in a mouse model of dry age-related macular degeneration. Oxid. Med. Cell Longev. 2019, 1479571. 10.1155/2019/1479571 31781321 PMC6875405

[B37] Shen N. Wang T. Gan Q. Liu S. Wang L. Jin B. (2022). Plant flavonoids: classification, distribution, biosynthesis, and antioxidant activity. Food Chem. 383, 132531. 10.1016/j.foodchem.2022.132531 35413752

[B38] Song H. P. Chen J. Hong J. Y. Hao H. Qi L. W. Lu J. (2015). A strategy for screening of high-quality enzyme inhibitors from herbal medicines based on ultrafiltration LC-MS and *in silico* molecular docking. Chem. Commun. (Camb) 51 (8), 1494–1497. 10.1039/c4cc08728c 25503795

[B39] Song H. P. Wu S. Q. Hao H. Chen J. Lu J. Xu X. (2016). A chemical family-based strategy for uncovering hidden bioactive molecules and multicomponent interactions in herbal medicines. Sci. Rep. 6, 23840. 10.1038/srep23840 27025397 PMC4812296

[B40] Strzalka W. Ziemienowicz A. (2011). Proliferating cell nuclear antigen (PCNA): a key factor in DNA replication and cell cycle regulation. Ann. Bot. 107 (7), 1127–1140. 10.1093/aob/mcq243 21169293 PMC3091797

[B41] Sydykov A. Mamazhakypov A. Maripov A. Kosanovic D. Weissmann N. Ghofrani H. A. (2021). Pulmonary hypertension in acute and chronic high altitude maladaptation disorders. Int. J. Environ. Res. Public Health 18 (4), 1692. 10.3390/ijerph18041692 33578749 PMC7916528

[B42] Tan X. H. Zhang K. K. Xu J. T. Qu D. Chen L. J. Li J. H. (2020). Luteolin alleviates methamphetamine-induced neurotoxicity by suppressing PI3K/Akt pathway-modulated apoptosis and autophagy in rats. Food Chem. Toxicol. 137, 111179. 10.1016/j.fct.2020.111179 32035215

[B43] Tang H. Gupta A. Morrisroe S. A. Bao C. Schwantes-An T. H. Gupta G. (2024). Deficiency of the deubiquitinase UCHL1 attenuates pulmonary arterial hypertension. Circulation 150 (4), 302–316. 10.1161/circulationaha.123.065304 38695173 PMC11262989

[B44] Ungurianu A. Zanfirescu A. Margină D. (2024). Exploring the therapeutic potential of quercetin: a focus on its sirtuin-mediated benefits. Phytother. Res. 38 (5), 2361–2387. 10.1002/ptr.8168 38429891

[B45] Vanhaesebroeck B. Guillermet-Guibert J. Graupera M. Bilanges B. (2010). The emerging mechanisms of isoform-specific PI3K signalling. Nat. Rev. Mol. Cell Biol. 11 (5), 329–341. 10.1038/nrm2882 20379207

[B46] Wang J. Li H. Xia T. Feng J. Zhou R. (2021). Pulmonary arterial hypertension and flavonoids: a role in treatment. Chin. J. Physiol. 64 (3), 115–124. 10.4103/cjp.cjp_25_21 34169916

[B47] Wang R. Pan J. Han J. Gong M. Liu L. Zhang Y. (2022a). Melatonin attenuates dasatinib-aggravated hypoxic pulmonary hypertension via inhibiting pulmonary vascular remodeling. Front. Cardiovasc Med. 9, 790921. 10.3389/fcvm.2022.790921 35402542 PMC8987569

[B48] Wang S. Sun X. Wang Z. Zhou S. Su S. Nan X. (2022b). Vanillic acid attenuates monocrotaline-induced pulmonary arterial hypertension by enhancing NO signaling pathways. Nat. Product. Commun. 17 (9), 1934578X221128411. 10.1177/1934578X221128411

[B49] Xiao P. T. Kuang Y. J. Liu S. Y. Xie Z. S. Hao J. H. Liu E. H. (2022). The antihyperlipidemic equivalent combinatorial components from peel of Citrus reticulata 'Chachi. J. Food Drug Anal. 30 (1), 77–87. 10.38212/2224-6614.3388 35647727 PMC9930996

[B50] Xue Z. Zhou M. Liu Y. Qin H. Li Y. Zhu Y. (2023). A modified Fangji Huangqi decoction ameliorates pulmonary artery hypertension via phosphatidylinositide 3-kinases/protein kinase B-mediated regulation of proliferation and apoptosis of smooth muscle cells *in vitro* and *in vivo* . J. Ethnopharmacol. 314, 116544. 10.1016/j.jep.2023.116544 37088239

[B51] Yang Y. Yin L. Zhu M. Song S. Sun C. Han X. (2021). Protective effects of dioscin on vascular remodeling in pulmonary arterial hypertension via adjusting GRB2/ERK/PI3K-AKT signal. Biomed. Pharmacother. 133, 111056. 10.1016/j.biopha.2020.111056 33378960

[B52] Zhang J. J. Mao M. Shao M. M. Wang M. C. (2024a). Therapeutic potential of natural flavonoids in pulmonary arterial hypertension: a review. Phytomedicine 128, 155535. 10.1016/j.phymed.2024.155535 38537442

[B53] Zhang R. Li Z. Liu C. Yang Q. Lu D. Ge R. L. (2022). Pretreatment with the active fraction of Rhodiola tangutica (Maxim.) S.H. Fu rescues hypoxia-induced potassium channel inhibition in rat pulmonary artery smooth muscle cells. J. Ethnopharmacol. 283, 114734. 10.1016/j.jep.2021.114734 34648900

[B54] Zhang X. Qi F. Gao W. Li Y. Yang H. Li P. (2025). A newly discovered bioactive equivalence of combinatorial components of Angong Niuhuang pill improves ischemic stroke via the PI3K/AKT axis. J. Ethnopharmacol. 343, 119453. 10.1016/j.jep.2025.119453 39922326

[B55] Zhang X. Yang Z. Su S. Nan X. Xie X. Li Z. (2023). Kaempferol ameliorates pulmonary vascular remodeling in chronic hypoxia-induced pulmonary hypertension rats via regulating Akt-GSK3β-cyclin axis. Toxicol. Appl. Pharmacol. 466, 116478. 10.1016/j.taap.2023.116478 36940862

[B56] Zhang Y. Cui Y. Deng W. Wang H. Qin W. Huang C. (2017). Isoquercitrin protects against pulmonary hypertension via inhibiting PASMCs proliferation. Clin. Exp. Pharmacol. Physiol. 44 (3), 362–370. 10.1111/1440-1681.12705 27873355

[B57] Zhang Y. Yu J. Zhang W. Wang Y. He Y. Zhou S. (2018). An integrated evidence-based targeting strategy for determining combinatorial bioactive ingredients of a compound herbal medicine Qishen Yiqi dripping pills. J. Ethnopharmacol. 219, 288–298. 10.1016/j.jep.2018.02.041 29572106

[B58] Zhang Z. Chen J. Su S. Xie X. Ji L. Li Z. (2024b). Luteolin ameliorates hypoxic pulmonary vascular remodeling in rat via upregulating K(V)1.5 of pulmonary artery smooth muscle cells. Phytomedicine 132, 155840. 10.1016/j.phymed.2024.155840 38941817

[B59] Zhao C. Le X. Li M. Hu Y. Li X. Chen Z. (2023). Inhibition of Hsp110-STAT3 interaction in endothelial cells alleviates vascular remodeling in hypoxic pulmonary arterial Hypertension model. Respir. Res. 24 (1), 289. 10.1186/s12931-023-02600-5 37978368 PMC10655391

[B60] Zhi K. Li M. Bai J. Wu Y. Zhou S. Zhang X. (2016). Quercitrin treatment protects endothelial progenitor cells from oxidative damage via inducing autophagy through extracellular signal-regulated kinase. Angiogenesis 19 (3), 311–324. 10.1007/s10456-016-9504-y 27017346

[B61] Zuo W. Liu N. Zeng Y. Xiao Z. Wu K. Yang F. (2021). Luteolin ameliorates experimental pulmonary arterial hypertension via suppressing hippo-YAP/PI3K/AKT signaling pathway. Front. Pharmacol. 12, 663551. 10.3389/fphar.2021.663551 33935785 PMC8082250

