# Supplementary material for: Bioactive compound combinations from Rhodiola tangutica alleviate pulmonary vascular remodeling in high-altitude pulmonary hypertension rats through the PI3K–AKT pathway
Source: Front Pharmacol. 2025 May 2;16:1582677. doi: 10.3389/fphar.2025.1582677 (PMC12081410; doi:10.3389/fphar.2025.1582677)
Supplement: Supplementary file 1 [file DataSheet1.docx]

Supplementary Material

# Supplementary Figures

**
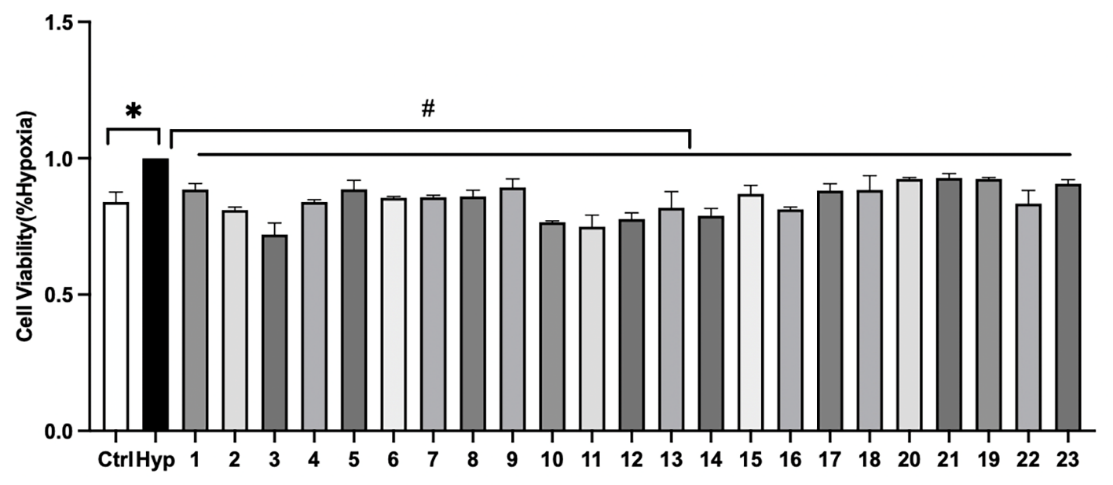
**

**Figure S1** The effect of the compounds from ACRT on anti-proliferation of hypoxia-induced PASMCs

Ctrl: Control; Hyp:Hypoxia; 1: Hyp+Arbutin-40μM; 2: Hyp+Eriodictyol-40μM; 3: Hyp+1,2,3,4,6-O-pentagalloylglucose-40μM; 4: Hyp+Saildroside-40μM; 5: Hyp+Rhodiosin-40μM; 6: Hyp+Rosiridin-40μM; 7: Hyp+Rosin-40μM; 8: Hyp+Crenulatin-40μM; 9: Hyp+Tyrosol-40μM; 10: Hyp+Epicatechin gallate-40μM; 11: Hyp+Quercitrin-40μM; 12: Hyp+Isoquercitrin-40μM; 13: Hyp+Epicatechin-40μM; 14: Hyp+Ferulic acid-40μM; 15: Hyp+Ethyl gallate-40μM; 16: Hyp+Kaempferol-40μM; 17: Hyp+P-Coumaric acid-40μM; 18: Hyp+Caffeic acid-40μM; 19: Hyp+Quercetin-40μM; 20: Hyp+Vanillic acid-40μM; 21: Hyp+Gallic acid-40μM; 22: Hyp+Luteolin-40μM; 23: Hyp+Catechin-40μM. *p < 0.05 *vs*. control group, ^#^p < 0.05 *vs*. hypoxia group.


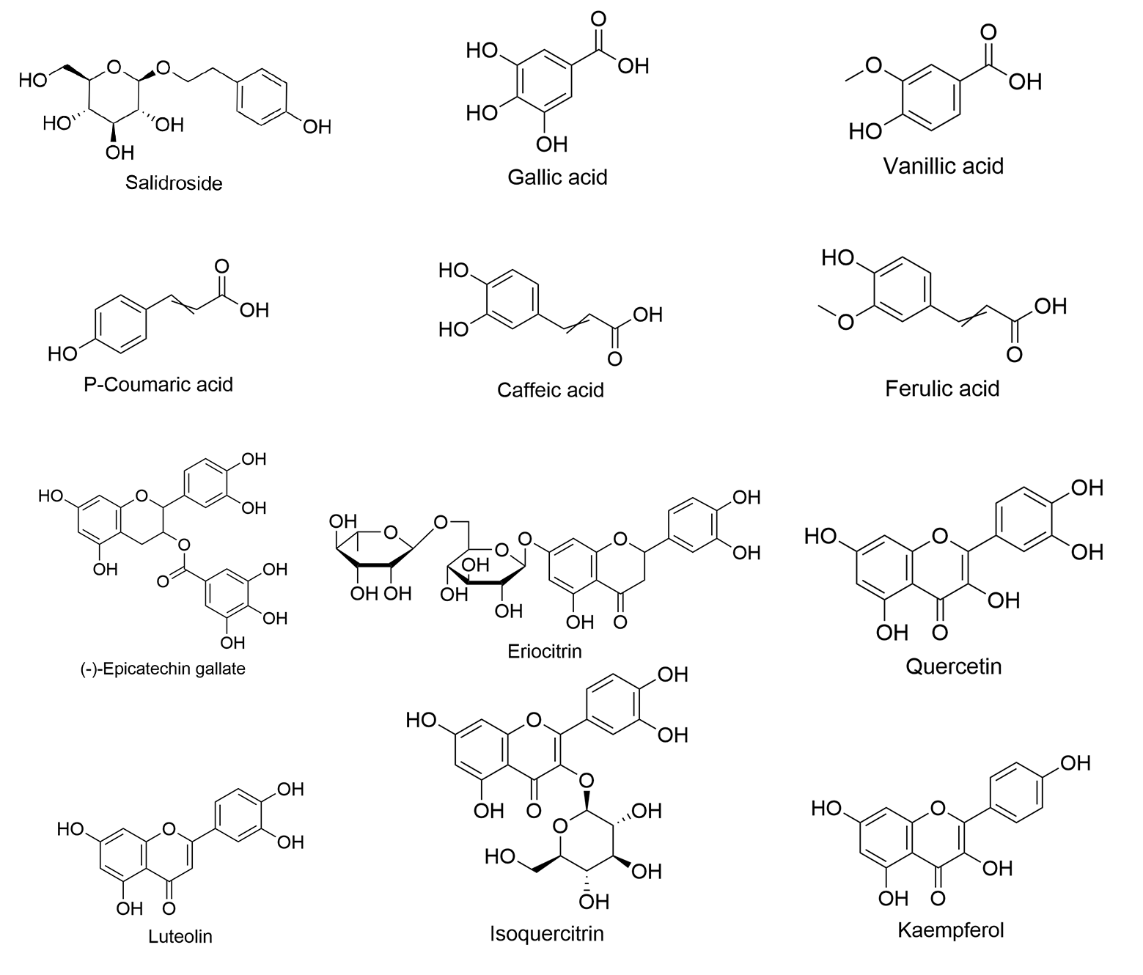


**Figure S2** Chemical structural of 12 compounds in BECCs


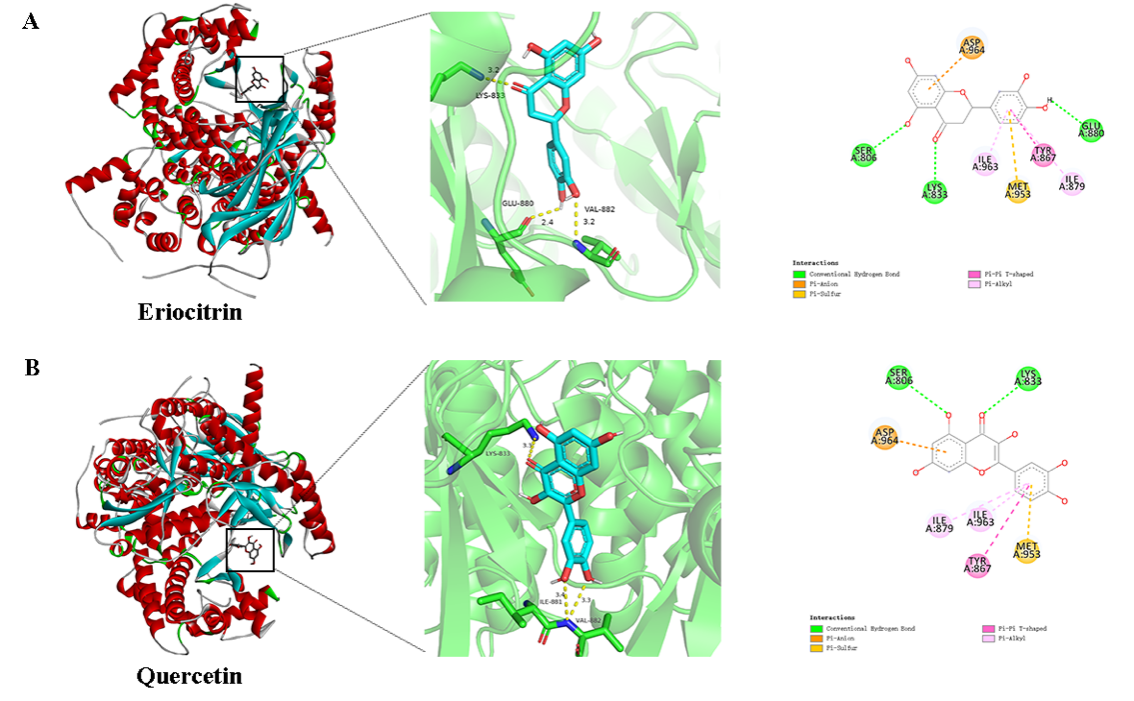


**Figure S3** The predicted model of the interaction between Quercetin or Eriodictyol and PI3K.

## 2 Supplementary Tables

**Table S1** The content of 12 abundant active ingredient in BECCs

| NO | Compound name | Content (μg/mg） | Combination |
| --- | --- | --- | --- |
| 1 | Salidroside | 48.63 | PeGs |
| 2 | Gallic acid | 27.18 | PA |
| 3 | Vanillic acid | 25.59 | PA |
| 4 | p-cumariacid | 3.60 | Phe |
| 5 | Caffeic acid | 2.34 | Phe |
| 6 | Ferulic acid | 1.26 | Phe |
| 7 | Eriocitrin | 4.02 | Fla |
| 8 | Kaempferol | 22.47 | Fla |
| 9 | Quercetin | 6.78 | Fla |
| 10 | Isoquercitrin | 6.21 | Fla |
| 11 | (-)-Epicatechin gallate e | 14.85 | Fla |
| 12 | Luteolin | 6.81 | Fla |

Fla :flavonoids, PeGs :phenylethanoid glycosides, Phe :Phenylpropanoids and PA :phenolic acids.

**Table S2. Effects of BECCs on body weight and organ coefficient in HAPH rat**

( $\bar{x}$± s ,n=10 )

| Group | body weight  （g） | liver  （mg/g） | spleen  （mg/g） | kidney  （mg/g） | lung  （mg/g） | heart  (mg/g） |
| --- | --- | --- | --- | --- | --- | --- |
| Control | 350.80±21.88 | 39.70±2.53 | 1.91±0.25 | 7.40±0.40 | 5.10±0.32 | 3.80±0.50 |
| Hypoxia (H) | 293.60±11.62* | 29.06±1.31* | 2.85±0.32* | 6.43±0.27* | 7.46±0.25* | 5.49±0.66* |
| H+ACRT  (150 mg/kg) | 301.60±10.84 | 34.40±1.05^#^ | 2.71±0.34 | 6.55±0.26 | 6.93±0.20^#^ | 4.99±0.47 |
| H+BECCs-L  (25 mg/kg) | 297.65±17.94 | 33.48±1.79^#^ | 2.68±0.39 | 6.55±0.16 | 6.75±0.31^#^ | 5.08±0.52 |
| H+BECCs-M  (50 mg/kg) | 302.60±9.98 | 34.34±1.45^#^ | 2.70±0.31 | 6.43±0.29 | 6.47±0.28^#^ | 4.85±0.60 |
| H+BECCs-H  (100 mg/kg) | 296.30±4.72 | 33.82±1.24^#^ | 2.59±0.24 | 6.37±0.25 | 6.73±0.38^#^ | 5.21±0.68 |
| H+ Sildenafil  (30 mg/kg) | 284.60±10.53 | 29.13±1.08 | 2.94±0.49 | 6.65±0.33 | 7.27±0.18^#^ | 4.94±0.61 |
| *F* | 3.17 | 36.32 | 1.23 | 1.52 | 17.86 | 1.49 |
| *P* | 0.0175 | <0.001 | 0.3058 | 0.1997 | <0.001 | 0.2072 |

ACRT: bioactive fraction from Rhodiola, 100 mg/kg; BECCs-L: Bioactive equivalent combinatorial components-25mg/kg; BECCs-M: Bioactive equivalent combinatorial components-50mg/kg; BECCs-H: Bioactive equivalent combinatorial components-100mg/kg/d; Sildenafil: 30mg/kg. ^*^*P* < 0.05 *vs.* control. ^#^*P* < 0.05 *vs.* Hypoxia.

**Table S3. Effects of BECCs on hematology related indicators in HAPH rat**

( $\bar{x}$± s ,n=10 )

| Group | HGB (g/L) | HCT (%) | RBC (10^^12^/L) | WBC (10^^9^/L) | PLT (10^^9^/L) |
| --- | --- | --- | --- | --- | --- |
| Control | 176.50±6.02 | 45.53±1.47 | 8.15±0.39 | 12.89±1.94 | 1015.00±91.07 |
| Hypoxia (H) | 252.00±6.91* | 69.45±1.37* | 10.58±0.41* | 9.18±0.83* | 582.60±49.07* |
| H+ACRT  (150 mg/kg) | 234.10±9.53^#^ | 64.93±2.46^#^ | 10.01±0.53^#^ | 11.45±1.13^#^ | 792.60±64.25^#^ |
| H+BECCs-L  (25 mg/kg) | 240.90±7.77^#^ | 67.00±2.11^#^ | 10.00±0.27^#^ | 11.72±2.01^#^ | 784.20±49.69^#^ |
| H+BECCs-M  (50 mg/kg) | 237.40±9.91^#^ | 67.35±2.71 | 9.90±0.45^#^ | 11.38±1.56^#^ | 761.20±53.90^#^ |
| H+BECCs-H  (100 mg/kg) | 224.30±11.51^#^ | 60.30±4.93^#^ | 9.165±0.53^#^ | 11.37±2.35^#^ | 746.60±80.67^#^ |
| H+ Sildenafil  (30 mg/kg) | 251.70±9.14 | 66.97±3.46 | 10.21±0.47 | 10.33±2.15 | 701.20±90.85^#^ |
| *F* | 13.30 | 10.58 | 10.74 | 3.06 | 13.78 |
| *P* | < 0.001 | < 0.001 | < 0.001 | 0.0168 | < 0.001 |

HGB: hemoglobin; HCT: hematocrit; RBC: red blood cell; WBC: white blood cell; PLT: platelet; ACRT: bioactive fraction from Rhodiola, 100 mg/kg; BECCs-L: Bioactive equivalent combinatorial components-25mg/kg; BECCs-M: Bioactive equivalent combinatorial components-50mg/kg; BECCs-H: Bioactive equivalent combinatorial components-100mg/kg/d; Sildenafil: 30mg/kg. ^*^*P* < 0.05 *vs.* control. ^#^*P* < 0.05 *vs.* Hypoxia.

**Table S4. Protein Identification**

| Title | Identified  peptides | Unique peptides | Identified proteins | Comparable  proteins |
| --- | --- | --- | --- | --- |
| Number | 38602 | 17173 | 5397 | 5395 |

**Table S5. Identification of phosphorylation modification sites**

| Title | Identified  sites | peptides | Modified  peptides | Identified  proteins | Comparable  sites | Comparable  proteins |
| --- | --- | --- | --- | --- | --- | --- |
| Number | 39648 | 16660 | 13394 | 10575 | 7985 | 2056 |
